# Prodomain processing controls BMP‐10 bioactivity and targeting to fibrillin‐1 in latent conformation

**DOI:** 10.1096/fj.202401694R

**Published:** 2025-02-08

**Authors:** Chara E. S. Spanou, Chengeng Yang, Alan R. F. Godwin, Stefanie Morosky, Arulselvi Anbalagan, Steffen Lütke, Matthias Mörgelin, Fady Marcous, Ubair Aziz, Alexander P. Wohl, Ishrat Jabeen, Manuel Koch, Thomas A. Jowitt, Beth L. Roman, Anna Tarakanova, Clair Baldock, Gerhard Sengle

**Affiliations:** ^1^ Department of Pediatrics and Adolescent Medicine, Faculty of Medicine and University Hospital Cologne University of Cologne Cologne Germany; ^2^ Center for Biochemistry, Faculty of Medicine University Hospital of Cologne Cologne Germany; ^3^ Department of Biomedical Engineering University of Connecticut Storrs Connecticut USA; ^4^ Wellcome Centre for Cell‐Matrix Research, Division of Cell Matrix Biology and Regenerative Medicine, School of Biological Sciences, Faculty of Biology, Medicine and Health, Manchester Academic Health Science Centre University of Manchester Manchester UK; ^5^ Department of Human Genetics, School of Public Health University of Pittsburgh Pittsburgh Pennsylvania USA; ^6^ Division of Infection Medicine, Department of Clinical Sciences Lund University Lund Sweden; ^7^ Colzyx AB Lund Sweden; ^8^ School of Interdisciplinary Engineering and Sciences National University of Science and Technology Islamabad Pakistan; ^9^ Institute for Dental Research and Oral Musculoskeletal Biology, Faculty of Medicine and University Hospital Cologne University of Cologne Cologne Germany; ^10^ Heart, Lung, Blood and Vascular Medicine Institute University of Pittsburgh Pittsburgh Pennsylvania USA; ^11^ School of Mechanical, Aerospace, and Manufacturing Engineering University of Connecticut Storrs Connecticut USA; ^12^ Center for Molecular Medicine Cologne (CMMC) University of Cologne Cologne Germany; ^13^ Cologne Center for Musculoskeletal Biomechanics (CCMB) Cologne Germany

**Keywords:** bone morphogenetic protein, complex, conformational change, electron microscopy, furin, growth factor, molecular dynamics, molecular modeling, proprotein convertases (PPCs), single particle analysis

## Abstract

Bone morphogenetic protein 10 (BMP‐10) is crucial for endothelial cell signaling via activin receptor‐like kinase 1 (ALK1), a pathway central to vascular homeostasis and angiogenesis. Dysregulated BMP‐10 signaling contributes to cardiovascular diseases and cancer, highlighting the need to control ALK1‐mediated endothelial responses to BMP‐10 for therapeutic development. BMP‐10 biosynthesis involves processing by proprotein convertases (PPCs) resulting in a non‐covalently associated prodomain–growth factor (PD–GF) complex (CPLX), similar to other TGF‐β superfamily ligands. However, the molecular requirements for BMP‐10 bioactivity remain unclear. We investigated how PPC processing impacts BMP‐10 structure, bioactivity, and its interaction with the extracellular matrix (ECM) protein fibrillin‐1. Molecular dynamics simulations post‐in silico cleavage of the BMP‐10 dimer model as well as negative staining and transmission electron microscopy (TEM) revealed that PD processing increases BMP‐10 flexibility converting it from a latent wide‐angle conformation to a bioactive CPLX which can adopt a V‐shape with tighter angle. Only processed BMP‐10 demonstrated high potency in HUVEC and C2C12 cells and robust binding to immobilized BMP receptors. Circular dichroism and interaction studies revealed that the N‐terminal region of the BMP‐10 PD is rich in alpha‐helical content, which is essential for efficient complexation with the BMP‐10 GF. Binding studies and TEM analyses showed that only the processed BMP‐10 CPLX interacts with the N‐terminal region of fibrillin‐1, causing a conformational change that renders it into a closed ring‐shaped conformation. These findings suggest that PD processing induces specific folding events at the PD–GF interface, which is critical for BMP‐10 bioactivity and its targeting to the ECM.

## Graphical abstract

Model of BMP‐10 prodomain‐growth factor conformation and its role in controlling bioactivity. The bioactivity of BMP‐10 is regulated through conformational changes governed by intracellular or extracellular processing by proprotein convertases (PPCs). This processing transforms BMP‐10 from an unprocessed, latent, and rigid open‐arm conformation to a processed, bioactive, and flexible conformation. In the bioactive state, BMP‐10 allows BMP types I and II receptors to access and bind to specific receptor‐binding sites on the growth factor (highlighted by yellow circles). The displacement of the prodomain during receptor binding occurs exclusively in the processed conformation, enabling activation of the growth factor. Only the processed BMP‐10 complex is capable of interacting with fibrillin‐1. This interaction targets BMP‐10 to the extracellular matrix (ECM), particularly to fibrillin microfibrils. Binding to fibrillin‐1 via the prodomain moiety induces a closed ring‐shape conformation, rendering the growth factor inaccessible and thereby maintaining BMP‐10 in a latent state within the ECM and ensuring spatial and temporal regulation of its activity.

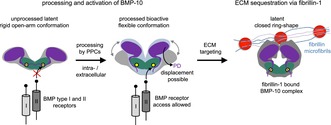

## 1 INTRODUCTION

Bone morphogenetic proteins (BMPs) belong to the TGF‐β superfamily of growth factors (GFs) and play pivotal roles in a multitude of biological events such as proliferation, differentiation, adhesion, and organogenesis.^1^ Similar to other TGF‐β family members, BMPs are secreted as complexes (CPLXs) comprising a GF dimer non‐covalently bound to two cognate prodomains (PDs).^2^, ^3^, ^4^, ^5^, ^6^ Both BMP moieties originate from a single precursor polypeptide chain. These precursors form dimers via disulfide bridges between specific cysteine residues in the two GF moieties. Processing by proprotein convertases (PPCs) results in C‐terminal PD cleavage right before the GF moiety at the consensus site: R/K‐Xn‐R/K↓^7^ to render the dimer into a processed non‐covalently associated PD–GF CPLX.

In recent years, several studies have demonstrated that the conformation of PDs of TGF‐β superfamily members defines the bioactivity of their cognate GFs, thereby acting as a protective shell to prevent unwanted signaling events.^8^ Interestingly, some members, such as GDF‐8 and TGF‐β, are cleaved by furin intracellularly and secreted in a latent form that requires additional extracellular proteolytic PD processing for GF activation.^9^, ^10^, ^11^, ^12^, ^13^, ^14^, ^15^ The additional activation mechanisms for TGF‐β1 GF have been investigated in more detail, the small latent PD–GF TGF‐β1 complex (SLC) was crystallized in a closed ring‐shape conformation, with both PD arms being disulfide‐linked within their C‐terminal regions.^11^ In this so‐called cross‐armed conformation the TGF‐β1 PD (also known as latency‐associated peptide: LAP) prevents access of TGF‐β receptors to the GF. In addition to proteolytical cleavage, TGF‐β GF activation involves several other mechanisms to remove the PD such as mechanical displacement via thrombospondin,^16^ or RGD‐site mediated integrin‐pulling.^11^, ^17^

Other TGF‐β superfamily members such as BMP‐7 and BMP‐9 are secreted in a bioactive V‐shape conformation, in which the PD competes with the BMP type II receptor ectodomain for the same GF binding sites, and is freely displaced upon receptor binding.^6^, ^18^, ^19^, ^20^ However, we previously showed that the BMP‐7 CPLX is converted from a bioactive open V‐shape into a latent closed ring shape upon binding to the extracellular microfibril component fibrillin‐1.^20^ Recently, we demonstrated an effective activation mechanism for BMP CPLXs from extracellular matrix (ECM) stored pools, mediated by specific degradation of BMP PDs by matrix metalloproteinases (MMPs).^8^, ^21^ However, not all V‐shaped CPLXs are bioactive in solution. Both unprocessed and furin‐processed GDF‐8 CPLXs are latent and adopt a V‐shape conformation.^12^ Processed GDF‐8 CPLX can be activated by tolloid‐like protein 2 (TLL2) cleavage of the PD (primed GDF‐8) and retains a V‐shape conformation.^9^

The molecular nature of secreted BMP‐10 remains unclear. Recent data suggest that BMP‐10 is predominantly a soluble CPLX expressed by hepatic stellate cells and cardiomyocytes that circulates in the bloodstream in association with PD.^22^ However, it is not known whether BMP‐10 is secreted in a processed or unprocessed form. Strong in vivo evidence indicates that unprocessed BMP‐10 is secreted and transported, as shown in human plasma where most BMP‐10 was detected in unprocessed form.^23^, ^24^ This suggests that BMP‐10 processing is mediated in a cell type‐specific manner by intracellular or extracellular furin, or other extracellular PPCs. It may be that depending on the cell type, BMP‐10 could be intracellularly folded, secreted, and then processed or intracellularly folded, processed, and then secreted. Accordingly, unprocessed BMP‐10 may be transported in an inactive form to target cells where it can be activated via membrane‐associated or extracellular PPCs. The conformation of BMP‐10 may therefore change during the transition from an unprocessed to a processed state.

While it is evident that the BMP‐10 PD associates with the GF in circulation, it is not clear how the PD affects signaling competency.^22^ Cell‐based assays have shown that titrating the PD to the BMP‐10 GF results in a cell‐type specific inhibition of its bioactivity. Specifically, adding the BMP‐10 PD at supraphysiological ratios quantitatively inhibited GF bioactivity in C2C12 myoblasts,^5^, ^25^ whereas this effect was not observed in human umbilical vein endothelial cells (HUVECs).^5^ However, it is still possible that the PD may confer latency in vivo. Like BMP‐7, the BMP‐10 PD was shown to interact with fibrillin‐1, suggesting that fibrillin microfibrils may also target BMP‐10 CPLXs to the ECM, thereby conferring latency.^22^, ^26^

Gaining a better understanding of the molecular requirements for BMP‐10 activity may lead to improved therapeutic approaches for cancer and vascular diseases. BMP‐10 is essential for cardiac development in mice and is thought to be implicated in the pathogenesis of congenital heart defects.^27^, ^28^, ^29^ Downregulation of BMP‐10 has been linked to cancer onset and progression, whereas its overexpression limits metastasis and prolongs survival.^30^, ^31^, ^32^, ^33^ Additionally, mutations in *BMP9* or the genes encoding BMP‐9/‐10 receptors, *ACVRL1* (encoding ALK1) and *ENG* (encoding endoglin), lead to hereditary hemorrhagic telangiectasia (HHT), a rare genetic disease that is characterized by arteriovenous malformations (AVMs) and internal bleeding.^34^, ^35^, ^36^ We delineated the roles of BMP‐9 and ‐10 in AVM development using zebrafish genetic models. While we found no requirement for *bmp9*, combined loss of duplicate *BMP10* paralogs, *bmp10* and *bmp10‐like*, results in embryonic lethal cranial AVMs indistinguishable from *acvrl1* mutants. Additionally, *bmp10* loss alone resulted in a later‐onset HHT‐like phenotype characterized by skin and liver vascular malformations and high‐output heart failure.^24^ These data, combined with a more recent genetic study in mice,^37^ strongly suggest that BMP‐10 is an indispensable ALK1 ligand necessary for vascular homeostasis and AVM prevention.

In this work, we aimed to study the structure and folding of full‐length BMP‐10 in processed and unprocessed forms and assess their respective bioactivities as well as their ability to be targeted to the ECM.

## 2 MATERIALS AND METHODS

### 2.1 Antibodies and proteins

For western blot and sandwich ELISAs the following antibodies were used: anti‐His_6_‐HRP (#130‐092‐785, Miltenyi Biotec, Germany), goat anti‐human BMP‐10 propeptide (#AF3956‐SP, R&D Systems, Minneapolis, MN, USA), mouse anti‐human BMP‐10 GF (#MAB2926, R&D Systems), rabbit anti‐human phospho‐SMAD 1/5/9 (pSMAD1/5/9) (#13820, Cell Signaling Technology, Danvers, MA, USA), mouse anti‐rabbit GAPDH (#ab8245, Abcam, Waltham, MA, USA), and rabbit anti‐human BMP‐7 GF (#500‐P198, PeproTech, Rocky Hill, NJ). Polyclonal rabbit anti‐fibrillin‐1 antiserum was raised against the C‐terminally His _6_ ‐tagged N‐terminal half of fibrillin‐1.^38^ Recombinant BMP‐10 GF was kindly provided by Andrew Hinck (Structural Biology, University of Pittsburgh).

### 2.2 Expression constructs

The cDNA sequences coding for the human BMP‐10 I314S/R315I variant with N‐terminal His_6_‐tag, full‐length human BMP‐10 with N‐terminal His_6_‐tag, and full‐length tag‐free human BMP‐10 were generated by gene synthesis (Genewiz, South Plainfield, NJ, USA), and cloned via NheI/BamHI sites into a pCEP‐Pu vector containing the signal peptide of BM40.^2^ Full‐length human BMP‐10 with a His_6_‐tag placed right before the furin cleavage consensus of the PD was generated by gene synthesis (Genewiz, South Plainfield, NJ, USA), and cloned via NheI/ BamHI sites into a sleeping beauty vector containing the signal peptide of BM40.

### 2.3 Cell culture

HEK293 EBNA cells were cultivated in DMEM GlutaMAX medium (#31966047, Thermo Fisher Scientific, Waltham, MA, USA) supplemented with 10% fetal bovine serum (FBS; #F7524‐500ML, Merck, Germany) and 1% penicillin–streptomycin (15140–130, Life technologies, Carlsbad, CA, USA). HUVECs (#C‐12208, PromoCell, Heidelberg, Germany) were cultured in complete endothelial cell growth medium 2 (EGM‐2, #C‐22111, PromoCell) containing 2% FBS. C2C12 cells were cultured in DMEM (D5796, Sigma–Aldrich, St. Louis, MI) supplemented with 10% FBS (#A3160401, Gibco, Thermo Fisher Scientific) and antibiotic/antimycotic solution (#SV30079.01, HyClone, Logan, UT, USA). Primary murine vascular smooth muscle cells (VSMCs) were isolated from aortic arches of male wild‐type mice as previously described^39^ and cultured in DMEM/F12 (#11320033, Thermo Fisher Scientific), 20% FBS, 1% penicillin–streptomycin, and 1× Smooth muscle growth supplement (#S00725, Gibco). All cells were maintained at 37°C in a 5% CO_2_, humidified incubator under sterile conditions.

### 2.4 Transient transfections and protein precipitation

cDNA constructs were transfected in HEK293 EBNA cells at 60% confluency in duplicates in 6‐well plates (#3516, Corning Costar, Corning, NY, USA) using the Fugene transfection reagent (#E2312, Promega, Madison, WI, USA). A 2 mL aliquot of the medium was collected after 24 h serum‐free conditions and TCA‐precipitated. The protein pellet was resuspended in 8 M ultrapure urea, 1 M NaCl in 20 mM NaH_2_PO_4_ × 2 H_2_O, pH = 7.4 after two ice‐cold acetone washes.

### 2.5 Co‐immunofluorescence staining of fibrillin‐1 and BMP‐10 in VSMC culture

Cells were cultured on poly‐d‐lysine‐coated coverslips at a density of 100 000 cells per coverslip and cultured under standard conditions for 7 days until confluency. The medium was replaced every 2 days. After reaching confluency, cells were maintained for an additional 5 days in fresh medium to promote ECM formation, followed by a 1‐day incubation with conditioned medium derived from HEK293 EBNA cells overexpressing full‐length BMP‐10 with the Sleeping Beauty transposon system. Coverslips were washed with PBS and fixed with ice‐cold methanol:acetone (1:1) at −20°C for 10 min. After fixation, cells were washed three times with PBS and blocked for 1 h at room temperature with 5% normal donkey serum (NDS) in PBS. Primary antibodies diluted in 0.5% NDS in PBS were applied overnight at 4°C. Coverslips were washed and incubated with fluorescently conjugated secondary antibodies in 0.5% NDS for 1 h at room temperature, followed by further washing. Coverslips were mounted using ProLong™ Diamond Antifade Mountant with DAPI (Thermo Fisher Scientific). Imaging was performed using a STELLARIS 5 microscope (Leica Microsystems) and analyzed with LAS X software.

### 2.6 Protein expression and purification

BMP‐7 CPLX was overexpressed and purified as previously described.^2^, ^20^ For BMP‐10, various constructs were transfected into HEK293 EBNA cells using Fugene: N‐terminally‐His_6_‐tagged BMP‐10 I314S/R315I mutant, tag‐free BMP‐10, N‐terminally His_6_‐tagged BMP‐10 with the endogenous furin site, and C‐terminally His_6_‐tagged BMP‐10. Cells were grown in triple flasks under puromycin selection (0.5–1 μg/mL for most constructs; 3 μg/mL for C‐terminally His_6_‐tagged BMP‐10) until reaching 85% confluency. Expression was induced with 0.5 μg/mL doxycycline. The C‐terminally His_6_‐tagged BMP‐10 was collected from the serum‐free medium supernatant. For purification of His_6_‐tagged BMP‐10 variants, 500 mL of conditioned medium were filtered (0.2 μm), pH adjusted to 7.4, and subjected to Ni‐NTA affinity chromatography (#74105, PureCube 100 Ni‐NTA Agarose, Cube Biotech, Germany). Elution fractions contained 5–250 mM imidazole concentration in 1 M NaCl, 20 mM NaH_2_PO_4_ × 2 H_2_O, pH 7.4. The purification of tag‐free BMP‐10 CPLX followed a revised protocol^5^: 500 mL of medium were filtered, pH adjusted to 7.6, and processed over a HiTrap Q HP column (#29051325, Cytiva, Marlborough, MA, USA). The column was washed with 20 mM Tris–HCl, pH 7.6, and additionally with 100 mM NaCl, 20 mM Tris–HCl, pH 7.6 until clear. Elution was performed with a NaCl gradient (100 mM to 2 M NaCl) in 20 mM Tris–HCl, pH 7.6. Fractions containing BMP‐10 were pooled and further purified using a second gradient elution on a HiTrap Q column. Pure BMP‐10 was then purified using size exclusion chromatography on a Superose 12 10/300 GL column (#GE17‐5173‐01, Cytiva) with a buffer of 1 M NaCl, 20 mM NaH_2_PO_4_ × 2H_2_O, pH = 7.4. Full‐length human BMP‐10, BMP‐9, and N‐10/C‐9 fusion PD sequences with a C‐terminal His_6_‐tag were expressed in *Escherichia coli* BL21‐CodonPlus Competent Cells (Agilent Technologies, Santa Clara, CA, USA) and purified by Ni‐NTA affinity chromatography, similar to *E. coli*‐derived BMP‐7 PD.^40^

### 2.7 Reconstitution assays

BMP‐10 PD and GF dimer (#C‐67317, Promocell) were mixed at a molar ratio of 2:1 in 20 mM HEPES, 0.8 M urea, pH 7.4, with 0.1% BSA as a carrier. The mixture was allowed to reconstitute in the presence of 2% casein with mild shaking at RT for 1.5 h. In a different setup, BMP‐10 PD and GF (#2926‐BP‐025/CF, R&D Systems) were placed in a mini dialysis device with a 2 kDa cut‐off (#69553, Thermo Fisher Scientific, Waltham, MA, USA) at a molar ratio 3:1 (PD: GF dimer) in the presence of 0.1% BSA. The samples were dialyzed stepwise into 4 M, 2 M, and 1 M urea in 1× PBS and plain 1× PBS, with each dialysis step lasting 4 h, followed by overnight dialysis into plain 1× PBS at 4°C. Next, the reconstituted material was subjected to size exclusion chromatography (SEC) on a Superose 12 in 1 M NaCl, 20 mM NaH_2_PO_4_ × 2 H_2_O, pH 7.4, to separate the PD–GF species from aggregated material or the PD dimer. The same dialysis procedure and SEC of a PD sample without added GF served as a control.

### 2.8 Sandwich ELISA

Sandwich ELISA assays were performed as previously described. Complex formation after reconstitution was assessed using a sandwich ELISA with a BMP‐10 GF antibody as the capture antibody and a BMP‐10 PD antibody as the detector. Nunc MaxiSorp flat‐bottom 96‐well plates (Thermo Fisher Scientific) were coated at 4 μg/mL in PBS overnight at 4°C. Coated surfaces were blocked with 5% casein in 20 mM HEPES, pH 7.4, followed by incubation with the reconstituted material in 2% casein in 20 mM HEPES, pH 7.4 for 2 h at RT. The wells were then washed three times with 20 mM HEPES, pH 7.4, followed by consecutive incubation with the detector antibody and secondary antibodies in 2% casein buffer. Signal development was carried out using 1‐Step Ultra TMB ELISA (#34028, Thermo Fisher Scientific), and the reaction was quenched with 10% sulfuric acid. Optical density (OD) was measured at 450 nm.

### 2.9 Stimulation assays with HUVEC and C2C12 cells

Cells were seeded in 6‐well plates and grown to 90% confluency over 2 days. HUVECs (passage 4–6) were serum‐deprived for 4 h in 0.2% FBS‐supplemented endothelial cell basal medium 2 (EBM‐2; #C‐22211, PromoCell) and treated for 45 min with 50 or 250 pg/mL growth factor equivalents of BMP10 GF, unprocessed BMP10 dimer (BMP‐10 I314S/R315I), processed BMP‐10 CPLX, or EBM2 + 0.2% FBS as a control. C2C12 cells were serum‐deprived for 4 h in 0.2% FBS‐supplemented DMEM and treated for 45 min with 4 or 20 ng/mL GF equivalents of BMP‐10 GF, unprocessed BMP‐10 dimer (BMP‐10 I314S/R315I), processed BMP‐10 CPLX, or 0.2% FBS + DMEM as a control. In each experiment, treatments were applied in duplicate and experiments were repeated 3 times.

### 2.10 Dot blot analysis

To examine the relevant fractions of reconstituted BMP‐10 CPLX after SEC for the presence of BMP‐10 PD and GF, fractions 17–33 were dot blotted on a nitrocellulose membrane, 0.45 μm (#10600002, VWR, Radnor, PA, USA), blocked with 5% milk in 1× TBS and then incubated with either a goat polyclonal BMP‐10 PD antibody or a mouse monoclonal BMP‐10 GF antibody. After incubation with the secondary antibody and several washes with 1× TBS‐Tween, the signal was developed using the Bio‐Rad Opti 4CN Substrate kit (#1708235, Bio‐Rad, Hercules, CA, USA). The intensity of obtained signals was quantified using ImageJ.

### 2.11 Western blotting

HUVEC or C2C12 cells were lysed in RIPA buffer (#89900, Thermo Fisher Scientific) with Halt Protease and Phosphatase Inhibitor Cocktail (#78440, Thermo Fisher Scientific) and frozen at −80°C. Just before use, samples were thawed, sonicated, and centrifuged. The cleared supernatants were collected, and protein concentrations were determined using the Pierce BCA Protein Assay (#23227, Thermo Fisher Scientific). Ten micrograms of protein were separated by 10% reducing SDS–PAGE and transferred to a nitrocellulose membrane (#1620115, Bio‐Rad). Membranes were dried for 1 h, rehydrated in water, and blocked in Intercept (TBS) Blocking Buffer (#92760001, LI‐COR, Lincoln, NE, USA) for 1 h. Antibodies were diluted in a blocking buffer with 0.1% Tween 20. Membranes were probed overnight at 4°C with a 1:1000 dilution of rabbit pSMAD1/5/9 antibody, followed by a 1:10 000 dilution of mouse GAPDH antibody for 1 h at room temperature the next day. Membranes were washed in TBS‐Tween and probed with 1:12 000 dilutions of IRDye 800CW donkey anti‐rabbit IgG (#925‐32213, LI‐COR) and IRDye 680LT goat anti‐mouse IgG (#926‐68020, LI‐COR) secondary antibodies for 1 h at room temperature, protected from light. Membranes were washed with TBS‐Tween while protected from light and imaged using the Odyssey CLx Imaging System (LI‐COR). pSMAD1/5/9 intensities were measured using Image Studio 5.2 software (LI‐COR) and normalized to GAPDH, according to manufacturer's instructions. The normalized signal values were then averaged for the technical replicates and divided by the 0.2% FBS medium control value to determine the fold‐change for each condition. This fold‐change was then graphed for each condition across each of the three independent assays using GraphPad Prism 8 (San Diego, CA, USA). To evaluate the inhibition of intracellular or extracellular furin processing, tag‐free full‐length BMP‐10 or BMP‐10 I314S/R315I elution fractions were subjected to SDS–PAGE using 15% gels, followed by western blot transfer to nitrocellulose membrane in 10 mM tetraborate buffer (131644, AppliChem, Darmstadt, Germany). The membrane was blocked with 5% milk in 1× TBS overnight at 4°C. The polyclonal goat BMP‐10 PD antibody was applied overnight in 2% milk in 1× TBS. After five washes with 1× TBS, the anti‐goat secondary was applied in 2% milk in 1× TBS, and signals were developed with the Bio‐Rad Opti 4CN Substrate kit. Quantification of signal intensities was performed using ImageJ. A similar western blot approach was used to detect BMP‐7 PD or GF in cell lysates or cell culture supernatants of BMP‐7 CPLX overexpressing HEK293 cells, but signals were developed using chemiluminescent substrates and X‐ray films in the dark. Cell lysates from BMP‐7 CPLX‐overexpressing HEK293 cells were obtained after two ice‐cold 1× PBS washes and resuspension of cells from an 80% confluent T‐75 flask in 1 mL RIPA supplemented with cOmplete EDTA‐free protease inhibitor cocktail (#11873580001, Merck). After a 10 min incubation on ice, the 1 mL cell suspension was collected in a microcentrifuge tube and centrifuged at 14 000 rpm for 20 min at 4°C. The supernatant from the lysed cell pellet was aliquoted and stored at −20°C before western blot analysis.

### 2.12 Single particle transmission electron microscopy

Unprocessed BMP‐10 I314S/R315I dimer was concentrated using a Q HP column and further purified over an s200i column twice in 1 M NaCl, in 1× PBS pH 7.4. The peak fraction was used to prepare negative stain EM grids as described previously.^21^ Images were collected on a Talos L120C G2 TEM at 120 keV with a magnification of 57 000×, a 1 s exposure, and a ~−1 μm defocus on a Ceta 16 M Camera. Automated particle picking was performed using a circular template with a Gaussian drop‐off “blob picker,” selecting 16 125 particles sized 100–200 Å. After 2D classification to exclude poor‐quality particles, 9181 particles were used to generate ab initio models. Good quality 2D class images served as templates for template‐based particle picking, which selected 87 068 particles. After excluding poor quality classes through 2D classification, 43 320 particles were used to refine the best 3D ab initio model. All particles picking and processing were performed using the cryoSPARC software.

### 2.13 Negative‐staining transmission electron microscopy and quantification

C‐terminally His_6_‐tagged BMP‐10 (unprocessed: processed 1:1) was adsorbed to negative‐staining EM grids for transmission electron microscopy (TEM). Samples were investigated in a Philips/FEI CM 100 electron microscope equipped with a tungsten emitter and a BioTWIN objective lens system at 80 kV accelerating voltage. Electron micrographs were taken with a side‐mounted Olympus Veleta camera with a resolution of 2048 × 2048 pixels (2 k × 2 K). To determine the percentage of molecules with wide or tight angles between the PD arms, 600 molecules were counted across 61 fields. For imaging closed‐ring BMP‐10, the N‐terminal fibrillin‐1 start‐EGF4 fragment^41^ was pre‐mixed with C‐terminally His_6_‐tagged BMP‐10 (unprocessed:processed 1:1) before adsorption to negative‐staining EM grids. Similarly, to assess the percentage of molecules with wide or tight angles between the PD arms after closed‐ring induction, 600 molecules were counted across 61 fields.

### 2.14 SEC/MALS and AUC

Two fractions of BMP‐10 were concentrated to 500 μL each and injected for SEC onto an S200 Increase column, operating at 0.6 mL/min in 1× PBS. Eluates were analyzed using 18‐angle Wyatt HeliosII and t‐REX detectors. For analytical ultracentrifugation (AUC), specific elution fractions post‐SEC/MALS were loaded into a two‐sector cell and centrifuged at 54 000 rpm. Scans were collected every 60 s at 280 nm. The sedimentation coefficients corrected for temperature and buffer conditions are 4.87 ± 0.15 and 6.17 ± 0.20 for the main peaks generated post‐AUC. The AUC data were fitted with a single species c(s) model fit^42^ with mass estimates based on an average frictional coefficient.

### 2.15 SPR binding studies

To assess the binding affinity of BMP‐10 processing variants to BMP receptors, the human IgG1‐Fc‐fusion ectodomains of BMPRII (#811‐BR‐100/CF, R&D Systems), ALK‐1 (#370‐AL‐100/CF, R&D Systems) as well as ENG (#1097‐EN‐025/CF, R&D Systems) were immobilized at 500 or 800 RUs on a CM5 chip via amine coupling. The analytes BMP‐10 GF (#2926‐BP‐025/CF, R&D Systems), BMP‐10 I314S/R315I (unprocessed BMP‐10 dimer), and processed BMP‐10 CPLX were injected in concentrations ranging from 0 to 80 nM in 1:2 serial dilutions in 1× HBS‐EP buffer. To assess the binding of the N‐10/C‐9 fusion PD to BMP‐10 GF, the PDs of BMP‐9 and BMP‐10 as well as the N‐10/C‐9 fusion PD were immobilized at 450 RUs on a CM5 chip via amine coupling. BMP‐10 GF was injected at concentrations from 0 to 80 nM in 1:2 serial dilutions using 1× HBS‐EP buffer. To evaluate the binding of BMP‐10 processing variants to fibrillin‐1, the fibrillin‐1 fragment start‐EGF4 was immobilized to 1200 RUs. The processing variants were then injected from 0 to 80 nM in 1:2 serial dilutions in 1× HBS‐EP buffer. All injections were in the HBS‐EP buffer. Kinetic constants were calculated by nonlinear fitting (1:1 interaction model with mass transfer) to the association and dissociation curves according to the manufacturer's instructions (BIAevaluation version 3.0 software). Apparent equilibrium dissociation constants (*K*_*D*_ values) were then calculated as the ratio of *k*_*d*_/*k*_*a*_.

### 2.16 CD spectroscopy

BMP‐10 PD (derived from *E. coli*‐ or 293HEK cells), the BMP‐10 I314S/R315I (unprocessed BMP‐10 dimer), and C‐terminally His_6_‐tagged BMP‐10 (processed BMP‐10 CPLX) were dialyzed into 5 mM HClO_4_ at 4°C. BMP‐9 PD and the N‐10/C‐9 fusion PD required dialysis into 20 mM HClO_4_ under the same conditions to maintain solubility. Human BMP‐9 CPLX (9624‐BP‐025/CF, R&D Systems) was also dialyzed into 20 mM HClO_4_ for consistency. CD spectra were recorded using a Jasco J‐715 spectropolarimeter in a 1 mm path length quartz cell (Hellma, Germany) from 260 to 170 nm at 20°C. BMP concentrations ranged from 0.05 to 0.1 mg/mL. Buffer contributions were subtracted, and theta/machine units were converted to Δε. The percentage of secondary structure was calculated using the CDSSTR algorithm, subset 3 (Dichroweb server). Theoretical CD spectra in Δε were obtained by submitting the relevant PDB files to the PDBMD2CD server (https://pdbmd2cd.cryst.bbk.ac.uk/).

### 2.17 Molecular modeling

To generate a model of the unprocessed BMP‐10 dimer, the AlphaFold prediction of the BMP‐10 monomer precursor (available at https://alphafold.ebi.ac.uk/entry/O95393) was duplicated and each monomer superimposed onto the BMP‐9 CPLX atomic structure (PDB: 4YCG). Specifically, each GF segment of the BMP‐10 precursor was aligned to the corresponding GF segment of the BMP‐9 CPLX structure to generate an unprocessed BMP‐10 dimer model.^43^ The BMP‐10 PPC cleavage sequence (RIRR) was then replaced with the PPC sequence from BMP‐7 (RSIR) and the model was refined by adding headers to introduce alpha helices within the BMP‐10 PD predicted by the experimental CD data, specifically residues E33‐V49 (part of α1‐helix), I157‐D159, D229‐S235, and S244‐H249. Steric clashes were resolved across the entire BMP‐10 structure, followed by a brief structural relaxation phase.

For validation of structural integrity and flexibility analysis, the V‐shaped model of processed bioactive BMP‐10 CPLX was simulated for a period of 100 ns in the GROMACS 2018 package.^44^ For this MD simulation, the structure was equilibrated using an NPT ensemble with TIP3P solvent model and CHARMM36m force field prior to the 100 ns simulation. Subsequently, the equilibrated unprocessed BMP‐10 dimer model was cleaved at the PPC cleavage site (RSIR) between the PD and the GF of each precursor of the dimer *in silico*, followed by a 200 ns simulation of the cleaved BMP‐10 dimer model. The processed model was selected from the trajectory based on its GF domain's similarity to the atomic model 7POI, with selection criteria guided by the lowest root‐mean‐square deviation of atomic positions. Headers were added indicating the same secondary structures as those in the crystal structure of BMP‐10 (PDB: 7POI) and BMP‐10 PD secondary structure map (Figure 5B) to the final structure of the processed BMP‐10 model.

To model the human BMP‐9 PD, covering α2 to α5 helices,^43^ the BMP‐9 PD structure was built on the 4YCG template using the PHYRE2 server.

For the closed‐ring BMP‐10 CPLX model, the BMP‐10 PD was modeled on TGF‐β1 precursor (PDB: 5VQF) using the SWISS‐MODEL server. The PD structure was adjusted to match the secondary structure mapping (Figure 5B), and docked as a receptor to the FUN domain of fibrillin‐1 (PDB: 2M74) as a ligand using the ClusPro 2.0 server. The resulting monomeric BMP‐10 PD‐FUN model (model 15) was reconstituted into a dimer by aligning two copies onto the closed‐ring BMP‐10 dimer model (PD covalently‐bound to GF also built on 5VQF) that was aligned on the closed‐ring BMP‐7 CPLX model.^21^, ^41^ Then, the BMP‐10 GF from the PDB structure 7POI was superimposed onto the GF of the closed‐ring BMP‐10 dimer model. The closed‐ring BMP‐7 CPLX and the closed‐ring BMP‐10 dimer were then deleted to reveal the processed closed‐ring BMP‐10 CPLX model. The docked model was then relaxed for 10 ns in an aqueous environment using the AMBER99‐ILDN force field in GROMACS 2023.3. Secondary structures were enforced based on those of BMP‐10 PD and GF using Modeller10.5. Sequence coverage for all models is detailed in Figure S7. All model manipulations were performed with the UCSF Chimera software^46^ unless otherwise specified. Chain‐by‐chain alignments were conducted using the “MatchMaker” tool within UCSF Chimera.

For distance measurements in BMP‐10 models, we used Visual Molecular Dynamics.^47^ In the unprocessed BMP‐10 model, residue E230, positioned at the tips of both PD arms, was selected for distance measurement. The distance between these residues on each arm reliably represents the overall width of the molecule. Similarly, in the processed BMP‐10 model, residue D176 on both PD arms was chosen for the same purpose, ensuring consistency in measuring the width across conformational states.

### 2.18 Surface charge calculations

To determine the surface charges of both the unprocessed BMP‐10 dimer and processed BMP‐10 CPLX models ChimeraX was employed.^48^ The Poisson Boltzmann Electrostatic Potential Surfaces were generated in Schrodinger v.12.6.117 at basic pH 8.4.

### 2.19 SASA measurements

To measure the solvent‐accessible surface area (SASA) of receptor binding pockets in the growth factor of unprocessed and processed BMP‐10, we utilized the built‐in function in the GROMACS simulation package,^44^ which implements Eisenhaber's algorithm.^49^ The receptor binding pockets for ALK‐1 and BMPRII were defined based on residues from BMP‐10 located within 10 Å of ALK‐1 or BMPRII, as inferred from crystal structures of ALK‐1 bound to BMP‐10 (PDB: 6SF3 and 7PPC), and BMPRII bound to BMP‐10 (PDB: 7PPA and 7PPC), respectively.

### 2.20 Statistical analysis

For quantitative western blot analysis, one‐way ANOVA with multiple comparisons was performed using GraphPad Prism 8.

## 3 RESULTS

### 3.1 Generation of unprocessed BMP‐10 dimer and processed BMP‐10 complex

**FIGURE 1:**
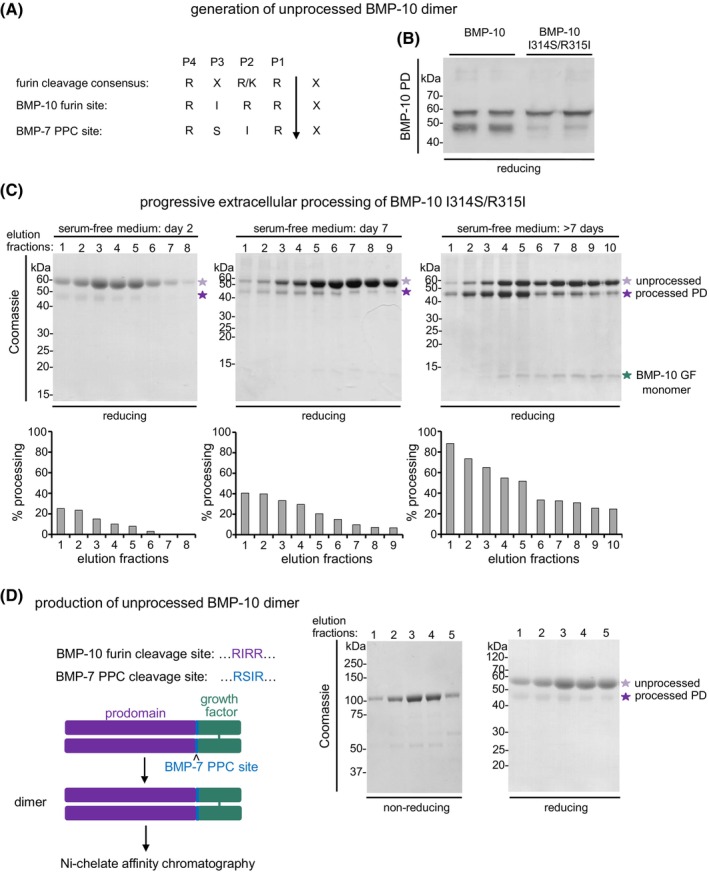
Generation of unprocessed BMP‐10 dimer. (A) Schematic representation illustrating the BMP‐10 and ‐7 PPC cleavage sites together with the furin cleavage consensus site. (B) Western blot analysis of BMP‐10 PD in HEK293 supernatants transfected with full‐length BMP‐10, or the BMP‐10 I314S/R315I mutant construct. (C) Progressive extracellular processing of the BMP‐10 I314S/R315I mutant after prolonged contact (2–7 days) with the cell layer in serum‐free conditioned medium. (Upper panel) Coomassie‐stained SDS–PAGE gels under reducing conditions after Ni‐NTA affinity chromatography of 500 mL medium collected after 2 days, 7 days, and more than 7 days in contact with the cell layer. (Lower panel) Fraction of processed BMP‐10 as a percentage of the total BMP‐10 signal was determined for each fraction by densitometric analysis of corresponding western blots using the anti‐BMP‐10 PD antibody. (D) (left) Schematic representation illustrating the substitution of the endogenous BMP‐10 furin site with the BMP‐7 PPC site. (right) Coomassie‐stained SDS gels under non‐reducing and reducing conditions after Ni‐NTA affinity chromatography of 500 mL of conditioned medium from HEK293 cells overexpressing the BMP‐10 I314S/R315I mutant.

To conduct a biochemical comparison of unprocessed and processed BMP‐10, we aimed to recombinantly express both forms. For designing an overexpression construct for unprocessed BMP‐10 we took several aspects into consideration. Previously, it was shown that furin has a preference for the presence of “R” in the P2 position of the PPC cleavage consensus.^50^, ^51^ We therefore hypothesized that substituting the endogenous BMP‐10 PD PPC cleavage site (RIRR↓) with the BMP‐7 PD PPC cleavage site (RSIR↓) (BMP‐10 I314S/R315I mutant) (Figure 1A) would inhibit intracellular BMP‐10 processing. This hypothesis is supported by western blot analysis of cell lysates from stably transfected cells, which showed that BMP‐7 processing does not occur intracellularly (Figure S1).

Western blot analysis of the conditioned medium 1 day post‐transfection revealed that intracellular processing was significantly reduced in the BMP‐10 I314S/R315I mutant compared to the control (Figure 1B). Consistent with the extracellular processing requirement of the BMP‐7 CPLX cleavage site, prolonged contact of the BMP‐10 I314S/R315I variant with the cell layer led to the processing of BMP‐10 that increased over time, thus yielding a mixed population of unprocessed and partially processed BMP‐10 molecules (Figure 1C). Non‐reducing SDS‐PAGE analysis of affinity purified, BMP‐10 collected after 2 days of contact with the cell layer detected a prominent band at 120 kDa, indicating the formation of unprocessed disulfide‐linked dimers between the precursor GF moieties with a trace amount of the processed BMP‐10 co‐purifying (Figure 1D).

**FIGURE 2:**
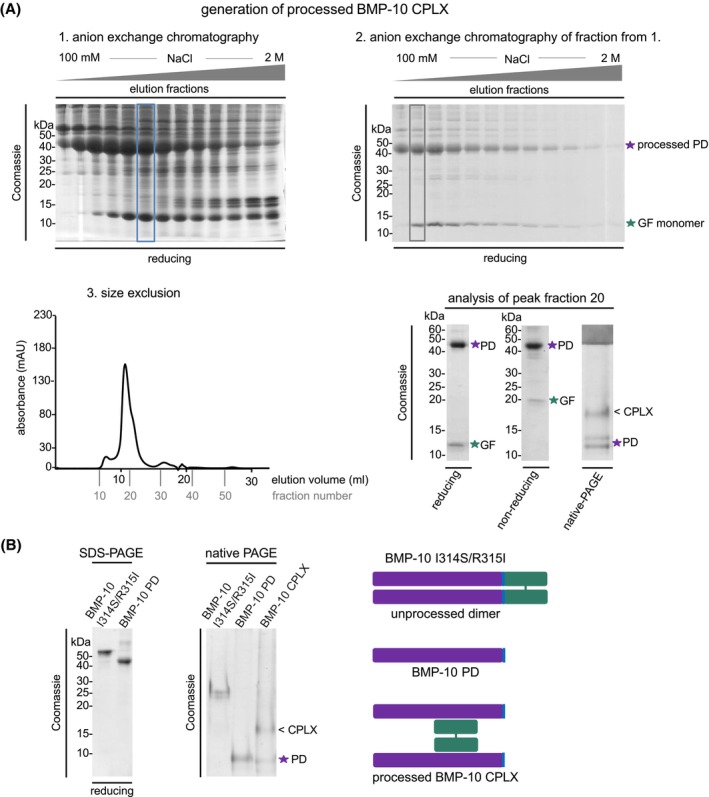
Generation and purification of the processed BMP‐10 CPLX. (A) 1. Coomassie‐stained SDS–PAGE gel of elution fractions containing BMP‐10 CPLX from the condensed conditioned medium after HiTrap Q HP anion exchange chromatography, 2. Coomassie‐stained SDS–PAGE gel of elution fractions after HiTrap Q HP anion exchange chromatography of the fraction highlighted in blue in 1. panel, 3. (left) SEC elution profile of processed BMP‐10 CPLX, (right) Coomassie‐stained SDS–PAGE gel after SEC purification of the fraction highlighted in gray in 2. panel, shown under reducing and non‐reducing conditions and native‐PAGE of the SEC purified peak fraction. Panel. (B) (left) Coomassie‐stained SDS–PAGE and native PAGE gels of BMP‐10 PD, the unprocessed BMP‐10 dimer, and the processed BMP‐10 CPLX. (right) Schematic representation illustrating the PD–GF arrangement in processed and unprocessed BMP‐10.

To recombinantly produce processed BMP‐10 CPLX, we generated a construct encoding full‐length BMP‐10 CPLX with its endogenous PPC site. Conditioned media from stably transfected HEK293 cells were concentrated over an anion exchange column (HiTrap Q) followed by salt gradient elution (Figure 2A1–2). Peak fractions were further purified by size exclusion chromatography (SEC) to remove high molecular weight aggregates, yielding intact BMP‐10 CPLX in fractions 19–21 (Figure 2A3). Under reducing conditions, the purified BMP‐10 complex (CPLX) exhibited bands at 45 kDa, corresponding to the PD with N‐glycosylation (calculated molecular weight without glycans: 34.6 kDa), and at 12 kDa, representing the monomeric growth factor (GF; calculated molecular weight of the monomer: 12.2 kDa; dimer: 24.4 kDa) (Figures 2A3, right and S2). Under non‐reducing conditions, the GF ran at 20 kDa due to the disulfide bond formation between GF monomers (Figure 2A3, right). Consistent with previous reports, the SEC‐purified BMP‐10 CPLX showed multiple bands on native‐PAGE, with the upper band corresponding to the fully processed BMP‐10 CPLX and lower bands indicating separated BMP‐10 PD^5^ (Figure 2A3, right).

Unprocessed and processed BMP‐10 migrated differently on native‐PAGE (Figure 2B), suggesting a change in net surface charge upon processing. Processed BMP‐10 CPLX displayed two bands after SEC in native‐PAGE, as shown previously.^5^ The lower bands migrated to the same position as the BMP‐10 PD, suggesting that the upper band corresponds to fully processed, non‐covalently associated BMP‐10 CPLX (Figure 2B).

### 3.2 Processed but not unprocessed BMP‐10 signals to both HUVEC and C2C12 cells

**FIGURE 3:**
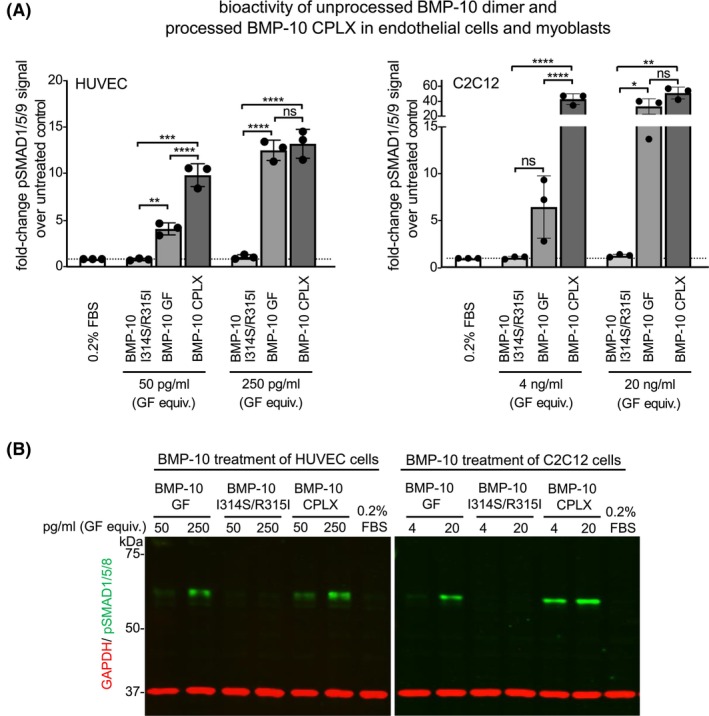
The processed BMP‐10 CPLX is bioactive towards HUVEC and C2C12 cells while the unprocessed BMP‐10 dimer is latent. (A) Quantification of western blot analysis for HUVEC and C2C12 cells treated for 45 min with the indicated BMP‐10 proteins. pSMAD1/5/9 intensity was normalized to GAPDH intensity, and values were expressed as fold change relative to 0.2% FBS control. Each data point represents the mean value of duplicates, derived from three independent experiments (*N* = 3). Error bars represent the overall mean ± SD. One‐way ANOVA with multiple comparisons. *p ≤ .05; **p ≤ .01; ***p ≤ .001; ****p ≤ .0001. (B) Representative western blots of cell lysates of HUVEC and C2C12 cells after 45 min stimulation with growth factor equivalents employing anti‐pSMAD1/5/9 and anti‐GAPDH antibodies.

To evaluate the bioactivity of unprocessed and processed BMP‐10, we stimulated HUVEC and C2C12 cells with GF equivalents of purified proteins (Figure S2) and quantified phosphorylation of SMAD1/5/9 via western blotting (Figure 3A,B). The unprocessed BMP‐10 dimer did not induce BMP signaling responses above baseline in either cell type (Figure 3A,B). By contrast, the processed BMP‐10 CPLX increased the level of pSMAD1/5/9 signals. At a concentration of 50 pg/mL, approximating the EC_50_ of the free BMP‐10 GF dimer, the BMP‐10 CPLX exhibited higher potency than the free GF dimer, whereas at higher concentrations the responses were similar (Figure 3A,B). In C2C12 cells, the unprocessed BMP‐10 dimer again failed to signal, while the processed BMP‐10 CPLX demonstrated higher potency near the EC_50_ (4 ng/mL) and similar potency at higher concentrations compared to the free BMP‐10 GF dimer. These results suggest that the previously observed inhibition of BMP‐10 GF activity by the PD in C2C12 cells^5^, ^25^ was due to supraphysiological PD concentrations. Additionally, at a physiologically relevant 2:1 ratio, the BMP‐10 PD does not inhibit GF activity in HUVEC or C2C12 cells (Figure 3A,B).

### 3.3 Processed but not unprocessed BMP‐10 binds to immobilized BMP receptor ectodomains

**FIGURE 4:**
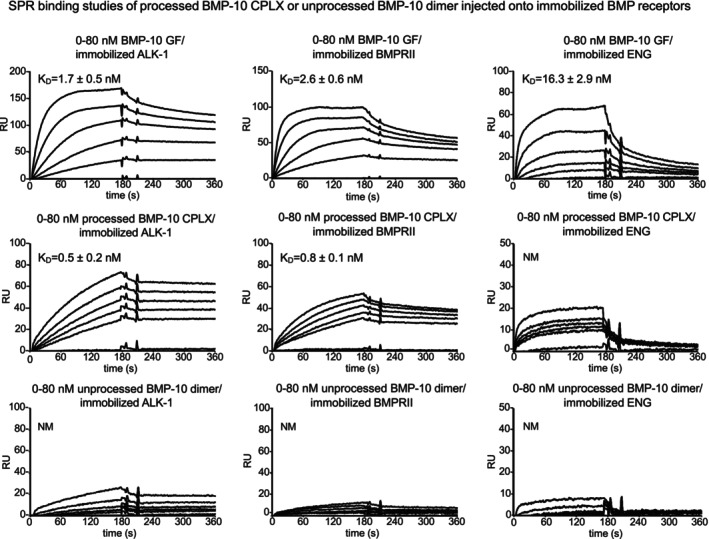
The processed BMP‐10 CPLX binds robustly to BMP receptors whereas the unprocessed dimer shows no receptor interaction. Sensorgrams of SPR interaction studies of soluble BMP‐10 GF, processed BMP‐10 CPLX, and unprocessed BMP‐10 dimer flowed over immobilized BMP receptors. Soluble analytes were injected onto immobilized BMPRII, ALK‐1, and ENG at concentrations ranging from 0 to 80 nM. *K*_*D*_s were calculated from three independent experiments (*N* = 3).

To investigate whether BMP‐10 processing enables BMP receptors to access the GF moiety, we conducted solid‐phase interaction studies using surface plasmon resonance (SPR). In this experiment, the ectodomains of BMP receptors BMPRII, ALK‐1, and endoglin (ENG) were immobilized, and either processed or unprocessed BMP‐10 was flowed over in increasing concentrations (0–80 nM), with BMP‐10 GF serving as positive control (Figure 4). Consistent with the bioactivity assay results in HUVEC and C2C12 cells, processed BMP‐10 showed higher affinity for the immobilized receptors compared to the free GF, whereas unprocessed BMP‐10 failed to bind (Table 1). The most striking reduction in receptor binding response of processed versus unprocessed BMP‐10 was observed for ALK‐1 and BMPRII, indicating that the BMP‐10 GF sites utilized by these receptors are inaccessible within the unprocessed dimer conformation (Figure 4 and Table 1). These findings are in line with a similar SPR binding study evaluating the binding capacities of both unprocessed and processed BMP‐9 to the same immobilized receptors.^18^

**TABLE 1:** SPR affinity data for interactions between the immobilized BMP receptors ALK1, BMPRII, and co‐receptor ENG.

Interaction (ligand/analyte)	*k*_on_ (1/M * s)/*k*_off_ (1/s)	*K*_*D*_ (nM)
ALK1/BMP‐10 GF	3.8 ± 1.3 × 10^5^/6.8 ± 4.1 × 10^−4^	1.7 ± 0.5
BMPRII/BMP‐10 GF	6.9 ± 1.8 × 10^5^/1.7 ± 0.1 × 10^−3^	2.6 ± 0.6
ENG/BMP‐10 GF	4.9 ± 0.6 × 10^5^/8.0 ± 2.4 × 10^−3^	16.3 ± 2.9
ALK1/processed BMP‐10	5.6 ± 0.1 × 10^5^/2.6 ± 1.0 × 10^−4^	0.5 ± 0.2
BMPRII/processed BMP‐10	9.4 ± 0.3 × 10^5^/7.9 ± 1.0 × 10^−4^	0.8 ± 0.1
ENG/processed BMP‐10	NM	NM
ALK1/unprocessed BMP‐10	NM	NM
BMPRII/unprocessed BMP‐10	NM	NM
ENG/unprocessed BMP‐10	NM	NM

Abbreviation: NM, not measurable.

### 3.4 The N‐terminal region of the BMP‐10 PD is required for interactions with the BMP‐10 GF and is rich in alpha‐helical content

Previous studies on BMP‐7 have shown that after processing, the PD assumes a stable secondary structure that remains unaltered even after separation from the GF allowing for the reconstitution of stable BMP‐7 CPLXs from separated moieties.^2^, ^20^, ^26^ The presence of an N‐terminal amphipathic α1‐helix containing hydrophobic residues is crucial for mediating specific interactions with the GF, contributing to high molecular affinity.^20^ A similar PD–GF interaction interface has been observed in the structural models of TGF‐β,^11^, ^52^ GDF‐8,^12^, ^53^ and pro‐activin.^54^ The BMP‐10 PD is predicted to contain an N‐terminal α1‐helix, similar to other TGF‐β superfamily members.^6^ However, it was recently proposed that BMP‐9 and BMP‐10 PDs adopt both conformations different from TGF‐β, GDF‐8, and pro‐activin within their processed CPLX forms, with the α5‐helix interacting with the GF, as seen in the atomic model of BMP‐9 CPLX.^43^

**FIGURE 5:**
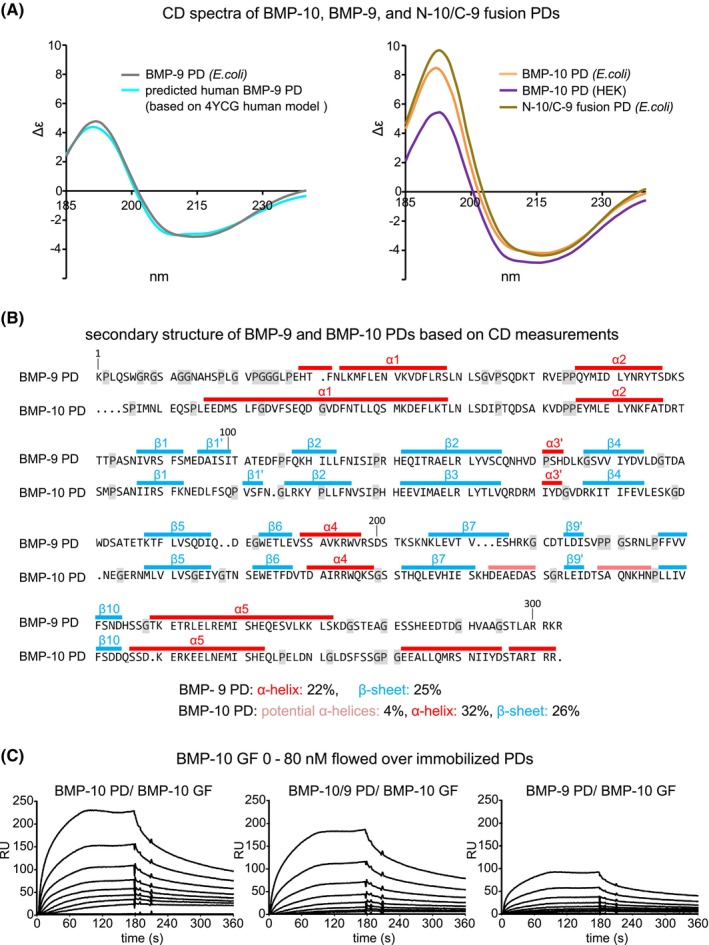
The N‐terminal region of the BMP‐10 PD containing the α1‐helix interacts with the BMP‐10 GF (A) CD spectroscopy analysis of BMP‐9 PD, BMP‐10 PD, and N‐10/C‐9 fusion PD expressed in *E. coli*. BMP‐10 PD obtained from BMP‐10 CPLX expression in HEK293 cells served as a control together with the *in silico* computed CD spectra of the BMP‐9 PD based on the atomic model 4YCG. (B) Secondary structure maps of BMP‐9 and BMP‐10 PDs were generated based on the obtained CD data. The position of α‐helices (red) and β‐sheets (*blue*) was guided by previously reported secondary structure predictions.^6^ Glycine and proline residues that are known to prevent the formation of alpha helices are marked in gray. BMP‐9 and BMP‐10 PD sequence alignment was conducted using the MultAlin online tool.^45^ (C) Sensorgrams from SPR binding studies of BMP‐10 GF flowed over immobilized BMP‐9 PD, BMP‐10 PD, and the N‐10/ C‐9 fusion PD at concentrations from 0 to 80 nM (representative sensograms of three independent experiments are shown).

To assess whether the recombinantly expressed PDs of BMP‐9 and BMP‐10 (Figure S3) assume a similar conformation, we analyzed their secondary structure by circular dichroism (CD) spectroscopy. The CD spectrum of the human BMP‐9 PD recombinantly expressed in *E. coli* closely matched the theoretically computed spectrum of the human BMP‐9 PD modeled from the murine structure (Figure 5A). The homology model of the human BMP‐9 PD was constructed using PHYRE2, using the atomic structure of the processed BMP‐9 CPLX (PDB: 4YCG) as a template, which includes the murine PD and the human GF sequence.^6^ The similarity in secondary structure indicates that similar to the BMP‐7 PD,^20^ the BMP‐9 PD can fold in the absence of its cognate GF. Both PDs adopt a similar conformation on their own or when complexed with their respective GFs2,6. The structure of the first 61 N‐terminal residues of the BMP‐9 PD were not resolved in the BMP‐9 CPLX structure.^6^ The high degree of overlay between the CD spectra of the bacterial‐expressed BMP‐9 PD and the N‐terminally truncated human BMP‐9 PD suggests that this N‐terminal stretch is unstructured and lacks α‐helical regions.

Our CD analysis revealed that the BMP‐10 PD has an α‐helical content of 36%–40%, which is almost twice as high as the 19%–22% observed for the BMP‐9 PD (Figure 5A and Table 2). The higher secondary structure content was consistent when comparing BMP‐10 PDs overexpressed in both *E. coli* and HEK293 cells (Table 2). The lower α‐helical content in the BMP‐9 PD compared to the BMP‐10 PD correlates with the higher amount of proline and glycine residues in the N‐terminal region of the BMP‐9 PD that limit α‐helix formation due to their destabilizing effects (Figure 5B). When we replaced the C‐terminal half of the BMP‐10 PD with the C‐terminal half of the BMP‐9 PD (N‐10/C‐9 fusion PD construct) (Figures S3 and S4), the resulting secondary structure was similar to that of the BMP‐10 PD, as evidenced by the similarity in their CD spectra (Figure 5A and Table 2). Guided by previous models^6^ and the measured secondary structure content (Table 2), we predicted secondary structure maps of the BMP‐9 and BMP‐10 PDs (Figure 5B). A comparison of these maps clearly shows a significantly shorter putative N‐terminal α1‐helix in the BMP‐9 PD compared to the BMP‐10 PD (Figure 5B).

**TABLE 2:** Experimental and theoretical secondary structure percentages of BMP PDs, GFs, and CPLXs used in this study.

BMP	α‐Helix	β‐Sheet	Turns	Unordered	Total
BMP‐10 PD (*E. coli*)	40	18	15	27	100
BMP‐10 PD (HEK293)	42	23	17	18	100
BMP‐10 I314S/R315I (HEK293)	31	22	19	28	100
Processed BMP‐10 CPLX (HEK293)	23	24	21	32	100
N‐10/C‐9 fusion PD (*E. coli*)	42	20	13	25	100
BMP‐9 PD (*E. coli*)	20	30	20	30	100
BMP‐9 CPLX (R&D systems)	16	31	20	33	100
BMP‐9 PD (PDB: 4YCG, theoretical)	19	38	7	36	100
BMP‐9 CPLX (PDB: 4YCG, theoretical)	18	40	8	34	100
BMP‐9 GF (PDB: 5I05, theoretical)	14	43	12	31	100
BMP‐10 GF (PDB: 6SF3, theoretical)	17	46	11	26	100

To test whether the BMP‐10 PD conformation readily allows BMP‐10 CPLX reconstitution, we dialyzed the *E. coli*‐derived BMP‐10 PD with the commercially available HEK293‐derived BMP‐10 GF in the presence of 0.1% BSA. The reconstituted sample was then subjected to size exclusion chromatography (SEC) followed by dot blot analysis (Figure S5A). Peak fractions showed the simultaneous presence of both PD and GF (Figure S5A), suggesting the successfully reconstitution of BMP‐10 CPLX molecules that elute in the same SEC fractions as the HEK‐derived processed BMP‐10 CPLX (Figure 2A3). Successful formation of the BMP‐10 CPLX was also demonstrated in the presence of 0.8 M urea using a sandwich ELISA (capture: anti‐GF, detector: anti‐PD) indicating robust and stable PD–GF assembly (Figure S5B). Interestingly, reconstituted BMP‐10 CPLX eluted in the same peak fractions as BMP‐7 CPLX after SEC (Figure S5C), suggesting that the reconstituted BMP‐10 CPLX has a similar hydrodynamic shape as the V‐shaped BMP‐7 CPLX.^20^

Previously, we determined the molecular affinity between the BMP‐10 PD and its GF to be in the low nanomolar range (*K*_*D*_ = 7 nM).^25^ To determine whether the N‐ or C‐terminal region of the BMP‐10 PD participates in GF complexation, SPR protein–protein interaction studies were performed. The results showed that the immobilized N‐10/C‐9 fusion PD interacted with the BMP‐10 GF with similar responses to the immobilized BMP‐10 PD, while the binding response to the immobilized BMP‐9 PD was considerably reduced (Figure 5C). This suggests that the extended α1‐helix within the N‐terminal region of the BMP‐10 PD is critical for efficient complexation with the BMP‐10 GF. Measurements of the association (*k*_*on*_) and dissociation (*k*_*off*_) rate constants revealed that the BMP‐9 PD and the N‐10/C‐9 fusion PD exhibit *k*_*on*_ rates approximately half as fast as the BMP‐10 PD (Table 3). This is likely due to a lower affinity binding site within the C‐terminal region of the BMP‐9 PD compared to that of the BMP‐10 PD, which contains the highly conserved α5‐helix. Consequently, the affinity constant (*K*_*D*_) for the BMP‐10 PD interaction with the immobilized BMP‐10 GF was determined to be lower (*K*_*D*_ ~ 5 nM), indicating stronger binding compared to the BMP‐9 PD and the N‐10/C‐9 fusion PD interactions (*K*_*D*_ ~ 10–13 nM).

**TABLE 3:** SPR affinity data for interactions between immobilized BMP‐9, ‐10, and ‐9/10 fusion PDs and flowed over BMP‐10 GF.

Interaction (ligand/analyte)	*k*_on_ (1/M * s)/*k*_off_ (1/s)	*K*_*D*_ (nM)
BMP‐10 PD/BMP‐10 GF	5.0 × 10^5^ ± 5.8 × 10^4^/2.4 × 10^−3^ ± 7.8 × 10^−5^	5.2 ± 0.7
BMP‐9 PD/BMP‐10 GF	2.3 × 10^5^ ± 6.6 × 10^4^/2.4 × 10^−3^ ± 7.1 × 10^−5^	10.4 ± 2.5
N‐10/C‐9 fusion PD/BMP‐10 GF	2.2 × 10^5^ ± 9.2 × 10^3^/3.2 × 10^−3^ ± 6.7 × 10^−4^	13.2 ± 3.5

### 3.5 Processing changes BMP‐10 conformation

**FIGURE 6:**
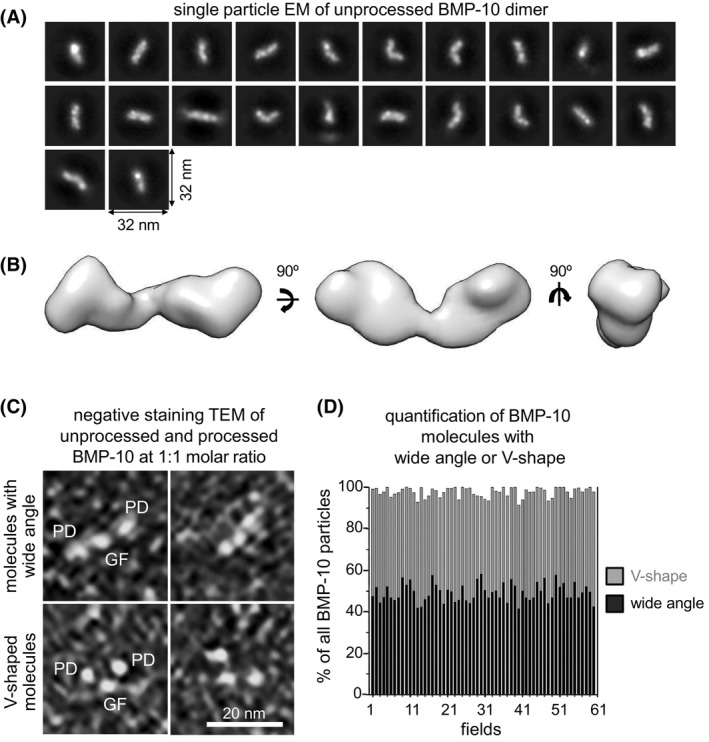
BMP‐10 CPLX assumes a V‐shape conformation while unprocessed BMP‐10 shows a conformation with a wider angle. (A) Single particle transmission EM class averages of the unprocessed BMP‐10 dimer from negatively stained images. (B) EM envelope of the 3D reconstruction of the unprocessed BMP‐10 dimer. (C) Negative staining TEM images of unprocessed and processed BMP‐10 at a 1:1 molar ratio, revealing BMP‐10 molecules with both V‐shape as well as with wide‐angle conformations. (D) Quantification based on 600 BMP‐10 CPLX particles per field across 61 different fields, showed an equal presence of molecules with a V‐shape and wide angle (1:1 molar ratio). Scale bar: 20 nm.

We analyzed the nanoscale structure of the unprocessed BMP‐10 dimer. Single particle TEM and subsequent 3D classification revealed a boomerang‐shaped conformation (Figure 6A,B). However, the initial purification of BMP‐10 provided only limited amounts of protein, which made further biochemical analysis difficult. To improve BMP‐10 production, we utilized the Sleeping Beauty transposon system in HEK293 cells. This system allows for stable integration of the BMP‐10 gene into the genome of cells, ensuring consistent expression of the protein. Additionally, we made the expression of BMP‐10 inducible by treating the cells with doxycycline, which can trigger the production of BMP‐10 when needed.^55^ Overexpressing full‐length BMP‐10 in this system resulted in the secretion of processed and unprocessed BMP‐10 in a 1:1 molar ratio, indicating an overwhelmed protein processing capacity of the cellular machinery (Figure S6). Subsequent analysis of this mixture using negative staining TEM, examining 600 particles across 61 fields, revealed that 50% of the molecules exhibited a wide angle and the other 50% a narrow angle (Figure 6C,D).

### 3.6 New models of unprocessed BMP‐10 dimer and processed BMP‐10 CPLX

**FIGURE 7:**
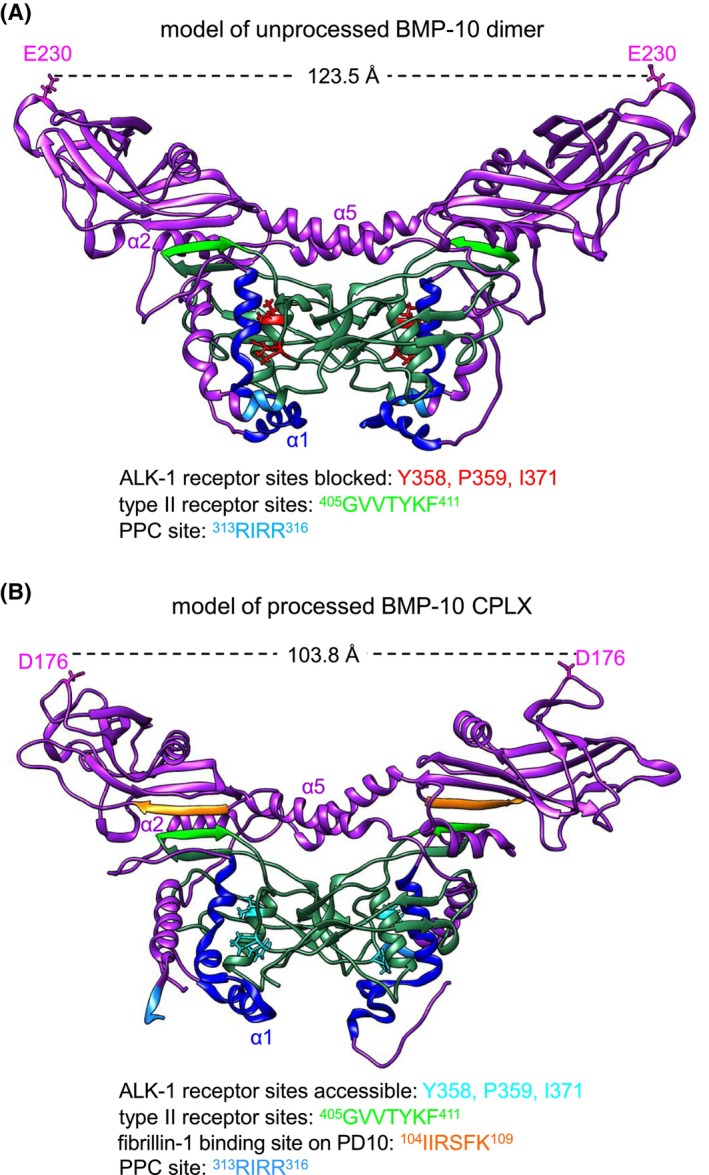
Models of unprocessed BMP‐10 dimer and processed BMP‐10 CPLX. (A) Model of the unprocessed BMP‐10 dimer showing the α1‐helix (marked in blue) masking ALK‐1‐binding residues Y358, P359, and I371 on the GF (marked in red). (B) Model of the processed BMP‐10 CPLX where the same GF residues (marked in cyan) are accessible for ALK‐1 receptor engagement. Processing reduces the distance between PD arms by about 20 Å as indicated. PPC cleavage sites are marked in light blue.

Next, we attempted to generate models of BMP‐10 in both its unprocessed and processed forms. A crystal structure of processed human BMP‐10 containing the PD and GF was recently deposited (PDB: 7POI). However, it is missing the first 57 amino residues which may contain the putative α1‐helix. Therefore, to construct a model of unprocessed BMP‐10, including these residues (Figure S7), we superimposed the monomeric human BMP‐10 precursor alphaFold model from the UniProt database onto the BMP‐9 (PDB: 4YCG) structure to generate a dimeric unprocessed BMP‐10 model. For this purpose, we chose the 4YCG structure in preference to the 7POI structure as it was previously suggested as a good model for BMP‐10^43^ and offers greater sequence coverage than 7POI. This approach produced a model consistent with the EM envelope determined for unprocessed BMP‐10 (Figure 6A,B). In this model, residues Y358, P359, and I371 of the BMP‐10 GF, known to interact with the ALK‐1 receptor, are obscured by the α1‐helix within the dimer, rendering them inaccessible for receptor engagement (Figure 7A). To generate the processed BMP‐10 CPLX model, we performed *in silico* cleavage at the PD PPC consensus site of the unprocessed dimer model. This yielded a processed CPLX model adopting a conformation with reduced distance between the PD tips similar to the BMP‐10 CPLX atomic structure (PDB: 7POI) (Figures 7B and S7). Importantly, in the processed CPLX model, the ALK‐1 binding residues within the GF are now accessible and poised for receptor engagement (Figure 7B). This is supported by increased SASA values measured for ALK‐1 and type II receptors in the processed BMP‐10 variant compared to the unprocessed form (Table 4). Additionally, greater distances were observed between the receptor binding pockets of the GF and the PDs in the processed BMP‐10 CPLX compared to the unprocessed BMP‐10 dimer (Figures S8 and S9).

**TABLE 4:** Solvent accessible surface area (SASA) of receptor binding pockets in the GF of BMP‐10 processing variants.

SASA (nm^2^)	Unprocessed BMP‐10 dimer	Processed BMP‐10 CPLX
ALK‐1	67.74	72.09
BMPRII	74.27	76.28

Furthermore, molecular dynamics simulations showed significantly increased root mean square fluctuation (RMSF) values for all BMP‐10 PD and GF residues upon processing, indicating enhanced flexibility of these residues (Figure S10A). Specifically, PD residues proximal to GF residues, which constitute BMP types I and II receptor binding sites, showed increased flexibility and exposure upon processing (Figures S9 and S10B).

Both the in silico‐generated unprocessed and processed BMP‐10 models include the putative α1‐ and α5‐helix of the BMP‐10 PD and illustrate self‐interaction of the BMP‐10 PDs within the CPLX structure. This correlates with the presence of an additional peak in ultracentrifugation experiments of the processed BMP‐10 CPLX, indicating the presence of stable BMP‐10 PD dimers (Figure S11).

### 3.7 Processing alters BMP‐10 surface charge

**FIGURE 8:**
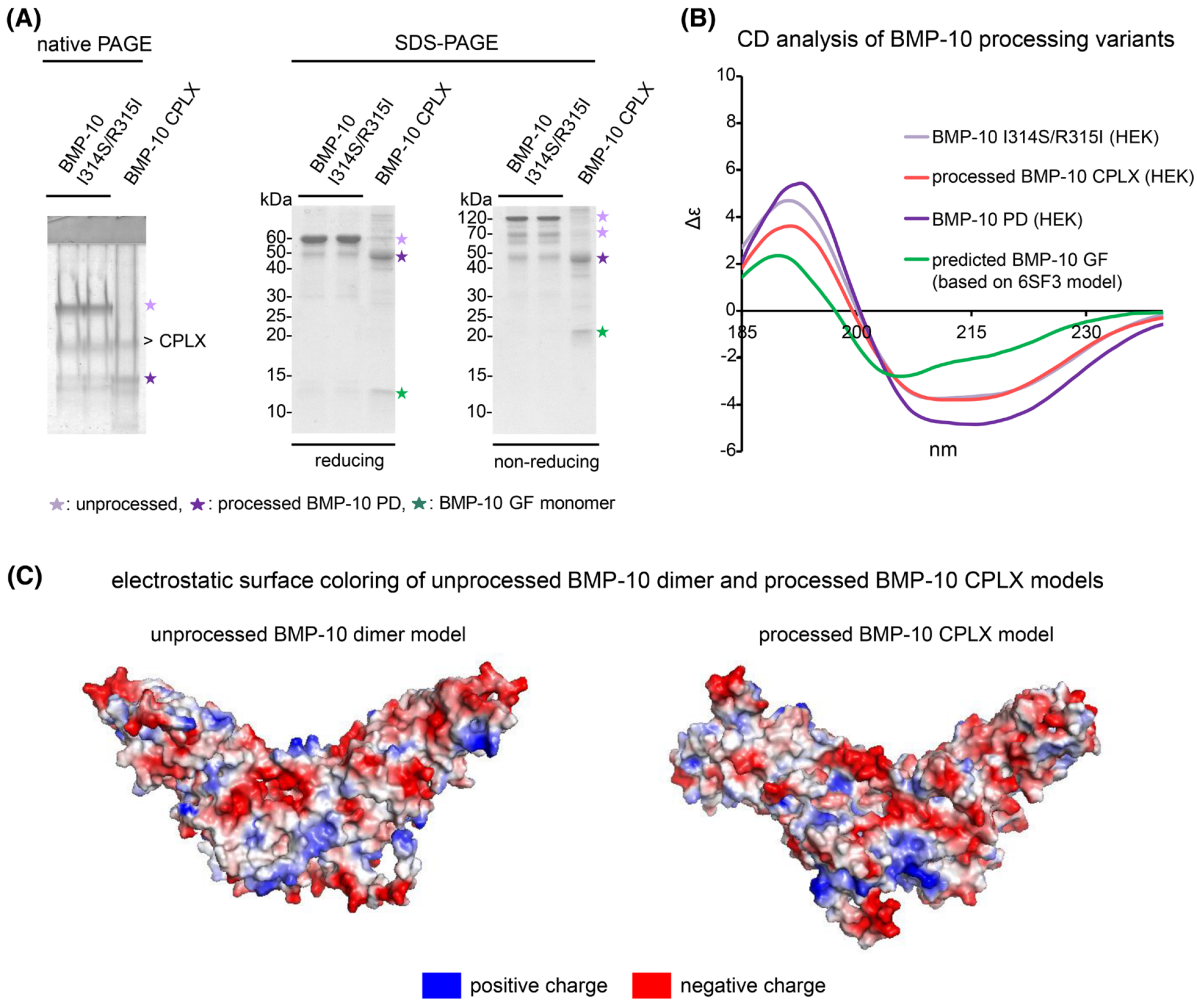
Prodomain processing alters BMP‐10 surface charge. (A) (left) Coomassie R‐stained native PAGE gel of unprocessed BMP‐10 I314S/R315I and processed BMP‐10 CPLX indicates different running behavior between the two BMP‐10 processing variants. (right) Coomassie‐stained SDS‐PAGE gels of unprocessed BMP‐10 I314S/R315I and processed BMP‐10 CPLX were analyzed under reducing and non‐reducing conditions. (B) Experimental and theoretical CD spectra of BMP‐10 PD, GF, unprocessed BMP‐10 I314S/R315I, and processed CPLX. (C) Electrostatic surface coloring of unprocessed BMP‐10 or processed BMP‐10 CPLX models using ChimeraX.

When subjected to native gel electrophoresis at pH 8.4, processed BMP‐10 CPLX migrated faster compared to the unprocessed BMP‐10 dimer (Figure 8A, left). CD measurements of BMP‐10 processing variants showed significant spectral overlap, indicating similar secondary structure content, thereby ruling out misfolding due to the BMP‐10 I314S/R315I point mutations (Figure 8B and Table 2). Therefore, we inferred that the difference in migration behavior in native‐PAGE is due to a change in surface charge upon processing. Indeed, analysis of surface charges revealed that processing changes the surface charges of BMP‐10 (Figure 8C). Computations of net surface charges using the Poisson Boltzmann method for the structural models of unprocessed and processed BMP‐10 at pH 8.4 showed that the processed BMP‐10 CPLX exhibits a higher negative surface charge (surface charge: −51) compared to the unprocessed BMP‐10 dimer (surface charge: −47) (Figure S12). This increased negative charge likely contributes to the accelerated migration of processed BMP‐10 in native PAGE.

### 3.8 Processing allows targeting of BMP‐10 CPLX to fibrillin‐1 in closed ring‐shape conformation

**FIGURE 9:**
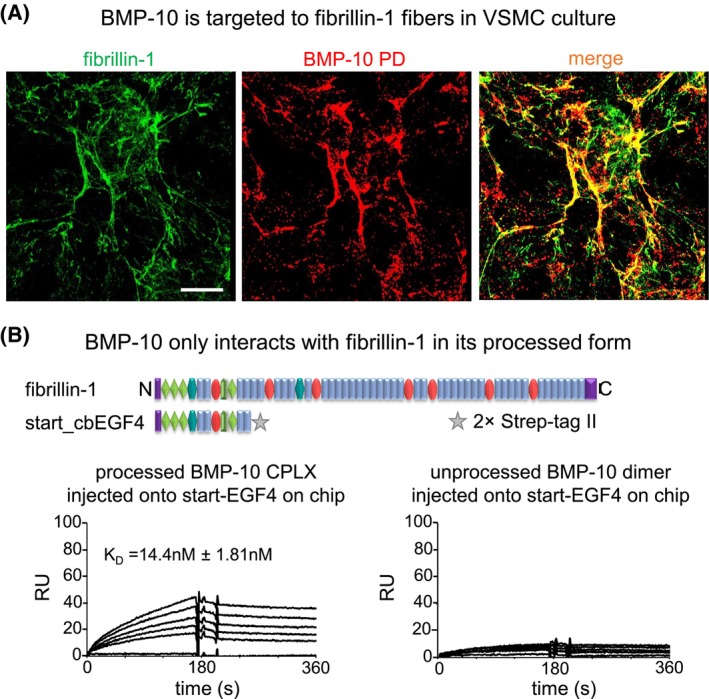
BMP‐10 is targeted to fibrillin‐1. (A) Co‐localization of BMP‐10 PD with deposited fibrillin‐1 fibers was detected in primary murine aortic VSMC culture. (B) SPR interaction experiment shows that only processed BMP‐10 interacts with the immobilized N‐terminal region of fibrillin‐1.

We previously showed that fibrillin‐1 interacts with the BMP‐10 PD suggesting that this is a targeting mechanism for the BMP‐10 CPLX ECM microenvironment.^25^ When we cultured primary murine vascular smooth muscle cells (VSMCs) in the presence of conditioned medium from BMP‐10 overexpressing HEK293 cells with the Sleeping Beauty transposon system, we observed co‐localization of BMP‐10 PD with fibrillin‐1 by confocal immunofluorescence microscopy (Figure 9A).

To investigate whether processing affects the targeting of BMP‐10 to the ECM we evaluated the binding of the unprocessed BMP‐10 dimer and the processed BMP‐10 CPLX to the N‐terminal region of fibrillin‐1 (Figure 9B). Using SPR, we immobilized the fibrillin‐1 fragment start‐EGF4 and flowed over BMP‐10 variants in solution. Interestingly, only the processed BMP‐10 CPLX showed a significant binding signal to immobilized fibrillin‐1, whereas the unprocessed BMP‐10 dimer exhibited no interaction (Figure 9B).

**FIGURE 10:**
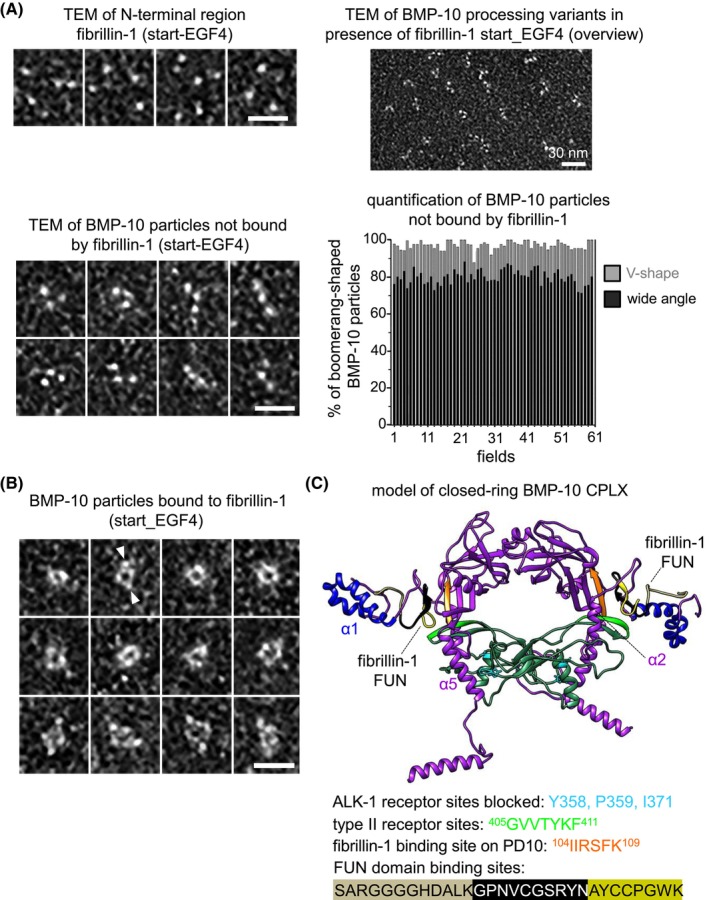
Processed BMP‐10 CPLX interacts with fibrillin‐1 and is rendered into a closed ring‐shape conformation. (A) TEM analysis after incubation of the N‐terminal fibrillin‐1N‐terminal region with a 1:1 mixture of processed and unprocessed BMP‐10. (top, left) Representative negative staining TEM images showing the globular shape of the N‐terminal region of fibrillin‐1 (start_EGF4). (top, right; bottom left) TEM analysis of unbound BMP‐10 molecules. Scale bars: 30 nm (overview) or 20 nm (magnified micrographs). (bottom, right) Quantification of unbound BMP‐10 particles showing a significant increase of molecules with wide angle (80%) versus tight angle (20%). (B) TEM analysis reveals the presence of ring‐shaped BMP‐10 molecules upon the addition of fibrillin‐1 (start_EGF4). The apparent position of globular N‐terminal fibrilin‐1 molecules is indicated by white arrow heads in one representative example. Scale bar: 20 nm. (C) Model of the closed‐ring BMP‐10 CPLX. In this model, the type II receptor binding site: ^405^GVVTYKF^411^ on BMP‐10 GF (light green) is masked by the α2‐helix, while ALK‐1 binding residues on the GF remain accessible. The BMP‐10 binding sites within FUN are labeled in black and yellow, and the fibrillin‐1 binding site in BMP‐10 PD is labeled in orange. PD residues are labeled in purple and GF residues in dark green.

To further explore whether the binding of BMP‐10 CPLX to fibrillin‐1 induces a closed ring‐shape structure similar to BMP‐7 CPLX,^20^ we incubated the 1:1 mixture of processed and unprocessed BMP‐10 with a four‐fold molar excess of fibrillin‐1 start‐EGF4 (total protein concentration 10–20 μg/mL) and subjected it to TEM analysis after negative staining. Start‐EGF4 alone displayed a globular shape approximately 5 nm in diameter (Figure 10A, left). However, upon mixing with unprocessed and processed BMP‐10 at a 1:1 ratio, ring‐shaped structures were observed, with globular molecules associated laterally (Figure 10B). The relative amount of molecules showing a ring‐shape was 22%. Upon closer inspection of 500 molecules, these ring‐shape structures were found to be targeted by two start‐EGF4 molecules (Figure 10B). Quantitative analysis of remaining boomerang‐shaped particles after the addition of fibrillin‐1 start‐EGF4 revealed an increase in the percentage of wide‐angled particles from 50% to 80% (Figure 10A, right). This suggests that the number of tight‐angled processed BMP‐10 molecules in the mixture is depleted upon binding to fibrillin‐1.

*In silico* molecular modeling and docking experiments enabled the generation of a closed ring‐shape model of BMP‐10 CPLX interacting with the FUN domain of fibrillin‐1 (Figure 10C). This model suggests that BMP‐10 PD residues ^104^IIRSFK^109^ interact with fibrillin‐1, while the GF is concealed within the interior of the ring. The ALK‐1 receptor binding residues Y358, P359, and I371 appear accessible for interaction, whereas the type II receptor binding site ^405^GVVTYKF^411^ is masked by the α2‐helix of the BMP‐10 PD, potentially blocking BMP signaling (Figure 10C).

To further investigate whether fibrillin‐1 binding stabilizes the closed ring‐shape conformation of processed BMP‐10, we conducted MD simulations both in the absence and presence of the fibrillin‐1 FUN domain. In the absence of FUN, the secondary structures of the processed BMP‐10 PDs exhibited reduced stability, leading to increased flexibility of the PD arms. This flexibility resulted in the transition from the closed ring‐shape conformation to an open V‐shaped conformation (see Video S1). In contrast, in the presence of fibrillin‐1 FUN, the secondary structures of the PD arms were maintained, restricting PD arm movement and stabilizing the closed ring‐shape conformation (see Video S2). Analysis of RMSF values derived from these MD simulations indicated significantly reduced flexibility of processed BMP‐10 PD and GF residues in the presence of the bound fibrillin‐1 FUN domain (Figure S13). Therefore, it can be concluded that this interaction with fibrillin‐1 FUN domain appears to stabilize the latent ring‐shape conformation of processed BMP‐10.

## 4 DISCUSSION

**FIGURE 11:**
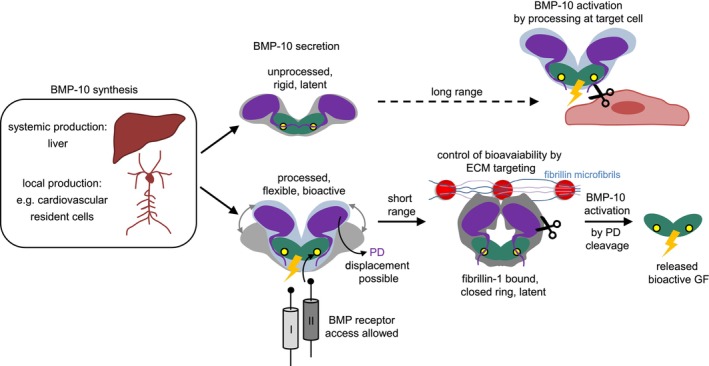
Working model describing targeting, sequestration, and activation of BMP‐10 depending on its processing status. BMP‐10 is produced systemically by hepatic stellate cells and locally by cardiovascular resident cells. Its activation depends on the localization of PPCs. When PPCs are present intracellularly, BMP‐10 is first processed and secreted in a bioactive form, which is then sequestered by fibrillin microfibrils in latent pools. From these pools, BMP‐10 can be reactivated locally through PD‐proteolytic cleavage, releasing the bioactive GF. Conversely, when PPCs are present extracellularly, BMP‐10 is secreted in an unprocessed, latent form. Activation occurs via PPC‐mediated cleavage of the covalent bond between the PD and the GF, which increases flexibility in the α1 helix, enabling specific GF residues to interact with ALK‐1 and initiate long‐range BMP signaling. These distinct activation pathways mediate either short‐range or long‐range signaling, activating unique signaling cascades during development and homeostasis.

In this study, we present models of full‐length BMP‐10 before and after PPC processing and demonstrate how this processing regulates BMP‐10 bioactivity. Specifically, we show that PPC processing increases flexibility at the PD–GF interface of the otherwise latent BMP‐10 dimer, allowing receptor access to the GF and rendering the CPLX signaling competent. Our results suggest a new working model for how BMP‐10 bioactivity is controlled by intra‐ or extracellular processing and sequestration via extracellular microfibrillar components, particularly fibrillin‐1 (Figure 11). BMP‐10 may be processed either intracellularly prior to secretion or extracellularly upon secretion, depending on the cell type. Secreted, processed bioactive BMP‐10 requires additional extracellular control of its bioavailability, while secreted latent BMP‐10 may be activated at the site of the target cell by specific PPC processing (Figure 11).

All TGF‐β superfamily members are processed at a conserved consensus motif (RXXR), but there is limited knowledge about whether this processing occurs within the cell or in the extracellular tissue microenvironment.^8^ Several members of the PPC family, PC1, PC2, furin, PC4, PC5, paired basic amino acid cleaving enzyme 4 (PACE4), and PC7, are essential for processing a broad array of precursor proteins which take place across various cellular compartments, including immature secretory granules, the *Golgi* apparatus, the cell surface, endosomes, and the ECM.^56^ However, the physiological conditions under which TGF‐β superfamily members are processed intra‐ or extracellularly remain poorly understood, particularly in terms of tissue‐specific and spatiotemporal regulation. There is evidence supporting the extracellular processing of TGF‐β superfamily members. For example, it was suggested that ECM proteins such as EMILIN‐1 may control extracellular processing of TGF‐β by furin convertases.^57^ Additionally, it was shown that GDF‐8 can be targeted to the extracellular muscle microenvironment in its unprocessed form.^58^ Since most BMP‐10 is found circulating in unprocessed form,^23^ its activation in tissues and cells with extracellular PPCs may occur after tissue‐specific targeting through interactions with ECM proteins. While BMP‐10 processing may share similarities with other TGF‐β superfamily members, the potential for tissue‐specific and ECM‐mediated interactions suggests the possibility of unique molecular requirements for its activation, which should be investigated in future studies.

Based on our previous findings that BMP‐10 PD interacts directly with fibrillin‐1,^26^ and that fibrillin‐1 binding induces a latent closed‐ring conformation in BMP‐7 CPLX,^20^ we hypothesized that ECM incorporation similarly regulates BMP‐10. In this study, we provide the first evidence that targeting BMP‐10 to fibrillin‐1 serves as a sequestration mechanism. Our results suggest that only processed BMP‐10 CPLX is targeted to supramolecular fibrillin‐1 fibers assembled by cardiovascular resident cells such as VSMCs (Figure 9A), while unprocessed BMP‐10 cannot. Our TEM negative staining (Figure 10B) and modeling results (Figure 10C) suggest that a latent ring‐shape is possible, wherein PD displacement is hindered due to interactions with fibrillin‐1. Similar closed‐ring models have been reported for processed BMP‐7 CPLX^20^ and BMP‐9 CPLXs.^6^ In the modeled closed conformation of the BMP‐9 CPLX, both the α1‐ and the α5‐helices are predicted to interact with the GF.^6^ In BMP‐10 CPLX, the PD α1‐ and α5‐helices likely position differently than in BMP‐9 CPLX.^6^ Therefore, it is plausible that BMP‐10 PD interactions with ECM proteins such as fibrillin‐1 help to preserve the closed ring‐shape conformation, inhibiting PD displacement and conferring latency. MD simulations of processed BMP‐10 CPLX in the presence and absence of the fibrillin‐1 FUN domain support this notion (Videos S1 and S2). These simulations indicate that the α2‐helix of the PD is stabilized in place by the FUN domain, blocking PD displacement and preventing BMPRII receptor binding to the GF. From latent ECM‐bound pools, BMP‐10 GF may be liberated through proteolytic cleavage of the PD (Figure 11), as we previously demonstrated for BMP‐7 GF.^21^ Our previous work suggests that BMP‐1^25^ and matrix metalloproteinases (MMPs) such as MMP‐2, ‐3, and ‐7 are likely involved in the specific and regulated degradation of the BMP‐10 PD.^21^

The targeting of BMP‐10 CPLXs by fibrillin‐1 may play a crucial role in modulating BMP‐10 activity, which in turn controls the contractile state of VSMCs.^59^ Although BMP‐10 CPLX circulates in the blood, it can reach the pericellular environment of VSMCs, potentially via transendothelial transport.^59^ BMP‐10 may also be secreted as a latent dimer and activated extracellularly by tissue‐specific PPC cleavage at lysine/arginine‐enriched consensus sites (R/K‐Xn‐R/K↓).^7^ Local PPC activity, such as from PACE4, may regulate BMP‐10 bioavailability in endothelial subsets, potentially contributing to HHT and enhancing the susceptibility to AVMs.^60^, ^61^ Structural differences between BMP‐9 and BMP‐10 (Figure 5) further underscore unique regulatory mechanisms despite their shared receptor profiles.^62^ Studies in zebrafish^24^ and mice^37^ highlight the predominant role of BMP‐10 in HHT progression, and furin‐mediated BMP‐10 processing may also influence hepatocellular carcinoma (HCC), a process potentially mitigated by BMP‐10 administration.^33^, ^63^

The presence of the PD at a physiologically relevant 2:1 ratio to the GF dimer may facilitate proper receptor targeting through interactions with unidentified cell surface molecules. For example, both C2C12 and HUVEC cells express the BMP co‐receptor endoglin (ENG) (GEO Profiles, Accession Numbers: 62009558 and 31844382), which contains a high‐affinity binding site for BMP‐9 GF. This site is conserved in the BMP‐10 GF sequence^64^ and can be used to displace the BMP‐9 PD.^65^, ^66^ According to our model of processed BMP‐10 CPLX, ENG binding to the GF would likely lead to PD displacement, as the predicted ENG binding region and the PD interaction site overlap on the BMP‐10 GF. Residues critical for ENG binding differ across BMPs with weak or absent ENG interactions,^67^, ^68^ potentially explaining differences in bioactivity observed between BMP‐7 and BMP‐10 CPLXs in C2C12 cells (Figure 3).^19^ Moreover, as C2C12 cells may express lower ALK‐1 levels than HUVECs, higher ligand concentrations could be required for effective signaling. At elevated BMP‐10 CPLX concentrations, the presence of the PD may enhance signaling compared to the GF alone (Figure 3), by stabilizing the GF in a bioactive state and increasing its spatial concentration on the cell surface. This is supported by our SPR binding studies, which show the enhanced affinity of the processed BMP‐10 CPLX to immobilized BMP receptors compared to free BMP‐10 GF (Figure 4 and Table 1).^65^ Interestingly, despite the different PD–GF interface described for the processed BMP‐9 CPLX and the reported weakened affinity of the BMP‐9 PD to its cognate GF,^6^ no enhanced bioactivity or receptor‐binding affinity was observed for the processed BMP‐9 CPLX compared to its GF alone.^6^, ^18^, ^65^ Consistent with our bioactivity and SPR binding results for unprocessed BMP‐10 (Figures 3 and 4), unprocessed BMP‐9 also failed to interact with immobilized BMP receptor ectodomains.^18^

Furin processing converts latent BMP‐10 dimers into a signaling‐competent BMP‐10 CPLX as shown in Figures 3 and 4. Unprocessed BMP‐10 dimers are unable to bind to ALK‐1, BMPRII, or ENG, (Figure 4), likely due to the α1‐helix of the PD masking key ALK‐1 binding sites on the BMP‐10 GF, as suggested by the model of the unprocessed BMP‐10 (Figure 7). Additionally, the covalent linkage between the PD and GF in the unprocessed BMP‐10 restricts PD flexibility, obstructing BMPRII and ENG receptor binding (Figures 4, 7, and S10). In contrast, upon cleavage, the α1‐helix undergoes repositioning (Figure 7), which exposes ALK‐1 binding sites on the GF and increases their solvent accessibility (Table 4). This processing significantly enhances PD flexibility (Figure S10), thereby increasing the distance between receptor‐binding pockets on the GF and PD compared to the unprocessed BMP‐10 (Figures S8 and S9). The non‐covalent PD–GF interaction in the processed CPLX permits PD displacement, which is essential for BMPRII and ENG receptor binding (Figures 4 and 7). We hypothesize that the slightly lower affinity of the processed BMP‐10 CPLX for ENG occurs because ENG functions as a co‐receptor and cannot displace the PD as effectively as BMPRII. Following PPC cleavage, processed BMP‐10 migrates faster on native gels than the unprocessed dimer (Figure 8A), due to an increased negative surface charge post‐cleavage (Figures 8C and S12), as both processing variants have a similar secondary structure (Figure 8B) and assume a boomerang shape (Figure 6A–C). Processing‐induced conformational changes could be observed via negative‐stain TEM (Figure 6C), and also found by measuring the distances between the tips of the PD arms in the processed and unprocessed models (Figure 7). Upon processing, BMP‐10 gains flexibility (Figure S10) and undergoes surface charge alterations (Figures 8C and S12), which likely contribute to its observed shift to a sharper conformation when analyzed by TEM after negative staining (Figure 6C,D). These conformational changes may be promoted by exposure to an acidic pH (around 4) or the binding of negatively charged uranyl acetate during the staining procedure. Modifications in surface charge can significantly affect the electrostatic interactions of processed BMP‐10, potentially destabilizing regions responsible for maintaining the original conformation with a wider angle. Consequently, BMP‐10 may adopt a structure with a sharper angle to achieve a more energetically favorable charge distribution similar to the reported V‐shape for BMP‐7 CPLX.^20^ This is consistent with the partial structure of the BMP‐10 CPLX (PDB: 7POI) which also displays a conformation similar to a V‐shape. Interestingly, our finding that processing of the latent BMP‐10 dimer results in a reduced distance between the tips of the PD arms in the bioactive CPLX is in line with previous reports on proactivin, where the unprocessed variant exhibits a more extended conformation with increased distance between PD arms and shows latency in bioactivity assays.^54^

SPR binding studies demonstrated a strong binding response between the N‐terminal region of BMP‐10 PD, containing the α1‐helix, and BMP‐10 GF, highlighting a robust PD–GF interface (Figure 5C). The observed differences in SPR responses among BMP‐10 PD, N‐10/C‐9 fusion PD, and BMP‐9 PD when interacting with BMP‐10 GF are likely due to the extended α1‐helix in BMP‐10 PD. Maximal binding responses occurred only when this α1‐helix was present, enabling dual‐site binding by stabilizing the interaction with the GF dimer. Interestingly, BMP‐9 PD displayed a slower association rate (*k*_*on*_) compared to BMP‐10 PD (Table 3), suggesting a weaker binding site in its C‐terminal region, possibly within the conserved α5‐helix, known to contribute to the PD–GF interface in the BMP‐9 CPLX.^6^ The N‐10/C‐9 fusion PD exhibited a similar slower on‐rate (Table 3), supporting the hypothesis that the weaker binding site resides in the BMP‐9 PD C‐terminal region.

PD–GF interfaces, where the α1‐helix of the PD plays a critical role, have been observed in proactivin,^54^ promyostatin,^12^, ^53^ and TGF‐β,^11^, ^52^ as well as previously by us in BMP‐7.^20^ Our previous interaction studies demonstrated strong PD–GF interfaces for BMP‐7, promyostatin, and BMP‐10 with binding affinities in the low nanomolar range (7–20 nM).^20^, ^25^ In contrast, the PD–GF interaction strength for BMP‐9 was measured to be two orders of magnitude lower (0.8 μM)^6^ than for BMP‐10 (5.2 nM, Table 3; 7 nM^25^), suggesting a less stable PD–GF interface and reduced CPLX stability for BMP‐9 compared to BMP‐10. In the BMP‐9 CPLX, the PD α5‐helix interacts with its cognate GF, assuming a different folding pattern from BMP‐10 PD, influenced by the presence of prolines and glycines in the amino acid sequence (Figure 5B). Unlike the processed BMP‐9 CPLX model, where no PD self‐interaction is observed,^6^ the processed BMP‐10 CPLX exhibits PD self‐interaction, as indicated by stable PD dimer detection in analytical ultracentrifugation experiments (Figure S11). This is similar to the processed BMP‐7 CPLX, for which receptor‐mediated PD displacement was demonstrated in velocity sedimentation experiments.^20^ In our in silico‐generated model of the processed BMP‐10 CPLX, this PD self‐interaction is mediated by the α5‐helix of each PD. This interaction mode excludes other hypothetical models where the BMP‐10 PD α5‐helix interacts with the BMP‐10 GF in a boomerang‐shape conformation, as such models do not predict BMP‐10 PD self‐interaction.

Overall, our data provide new insight into the molecular requirements for BMP‐10 CPLX bioactivity. Our findings may inform the development of new therapeutics for BMP‐10‐associated diseases like HHT and cancer.

## AUTHOR CONTRIBUTIONS

Chara E. S. Spanou and Gerhard Sengle conceived the study. Chara E. S. Spanou, Chengeng Yang, Alan R. F. Godwin, Stefanie Morosky, Arulselvi Anbalagan, Steffen Lütke, Matthias Mörgelin, Fady Marcous, Alexander P. Wohl, Manuel Koch, Ubair Aziz, Ishrat Jabeen, and Thomas A. Jowitt performed research and analyzed data. Matthias Mörgelin, Alan R. F. Godwin, and Clair Baldock conducted and evaluated EM analysis. Stefanie Morosky, Arulselvi Anbalagan, and Beth L. Roman, designed, performed, and evaluated bioactivity assays. Ubair Aziz and Ishrat Jabeen performed and analyzed MD simulations. Anna Tarakanova, Beth L. Roman, and Clair Baldock provided expert advice and edited the manuscript. Chara E. S. Spanou and Gerhard Sengle wrote and edited the manuscript. Anna Tarakanova, Beth L. Roman, Manuel Koch, Clair Baldock, and Gerhard Sengle acquired funding for this study.

## DISCLOSURES

The authors declare no conflict of interest.

## Supporting information


Figure S1.

Video S1: MD simulation of processed BMP‐10 CPLX.

Video S2: MD simulation of processed BMP‐10 CPLX bound to the N‐terminal unique domain of fibrillin (FUN).

## Data Availability

The data that support the findings of this study are available in the Materials and Methods, Results, and/or Supplemental Material of this article.
