# Supplementary material for: Prodomain processing controls BMP‐10 bioactivity and targeting to fibrillin‐1 in latent conformation
Source: FASEB J. 2025 Feb 8;39(3):e70373. doi: 10.1096/fj.202401694R (PMC11806408; doi:10.1096/fj.202401694R)
Supplement: Supplementary file 1 — Figure S1. [file FSB2-39-e70373-s003.pdf]

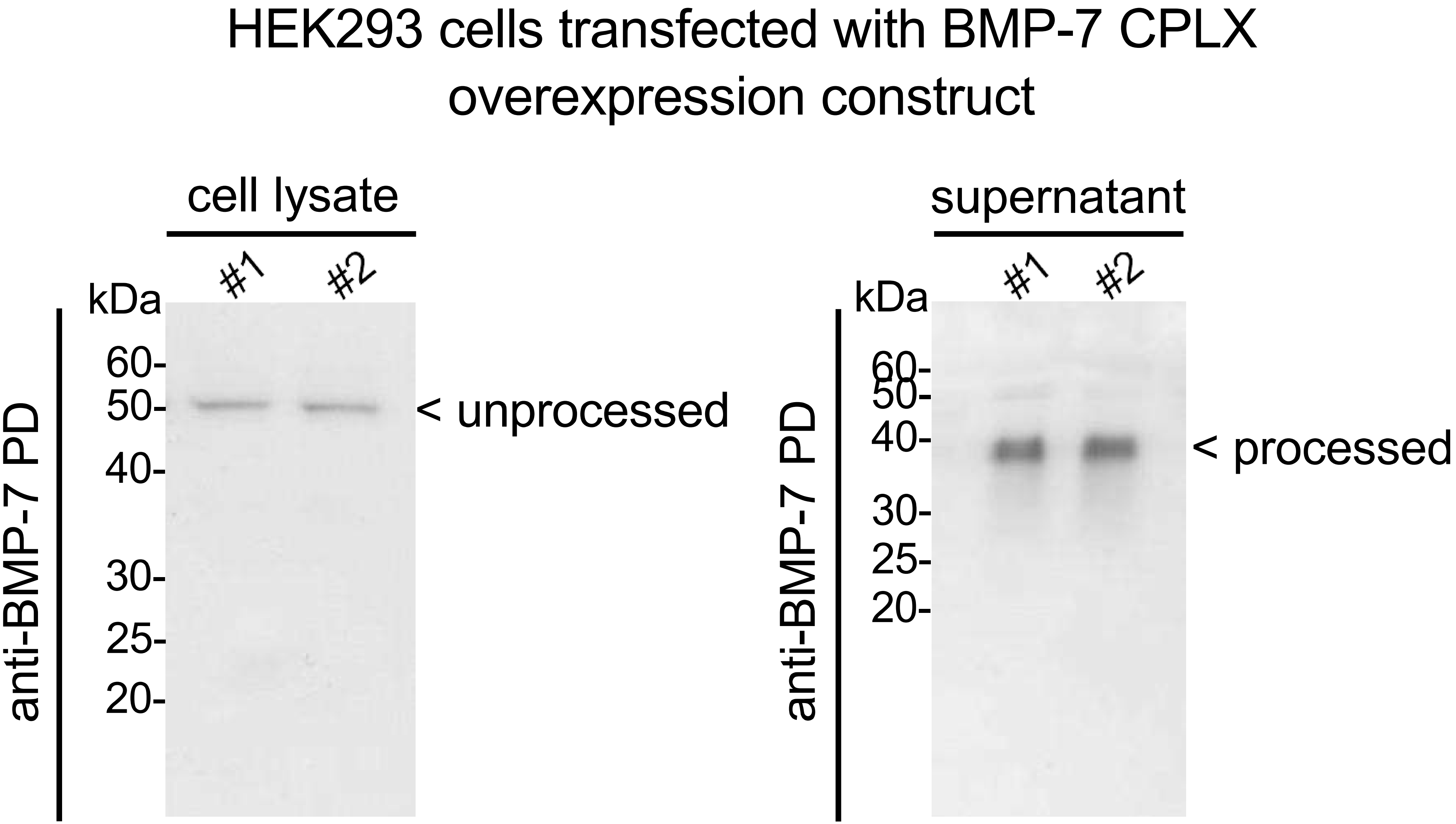

Figure S1: BMP-7 CPLX is processed extracellularly. Transfection of HEK293 cells with full length BMP-7 CPLX construct shows extracellular processing of BMP-7 PD. Western blot analysis of cell lysates and conditioned media was performed with monoclonal anti-BMP-7 PD mab2 and mab33 (1:1 mixture) and polyclonal anti-BMP-7 GF (Peprotech).

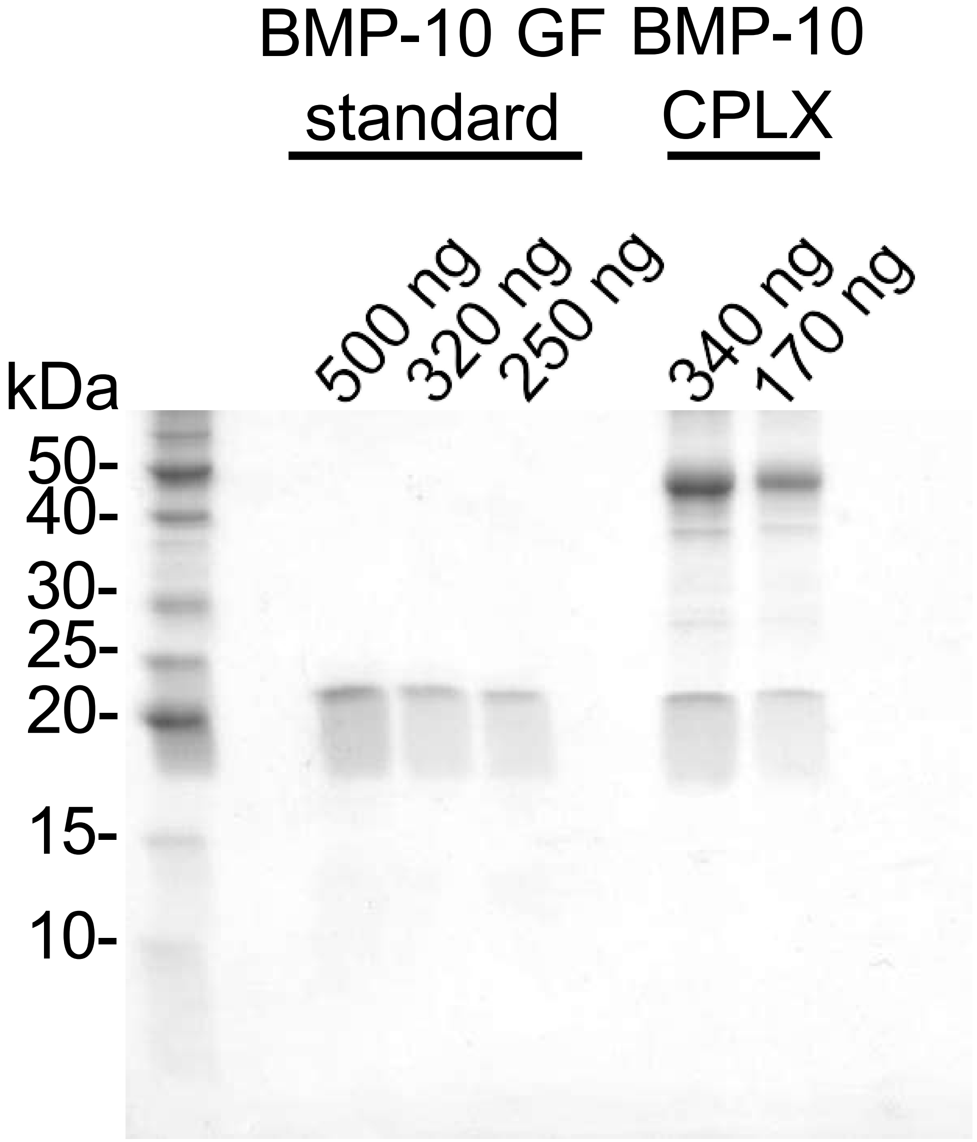

Figure S2: Representative quantification of BMP-10 GF amounts in preparations of processed BMP-10 CPLX. Human BMP-10 GF (Promocell) was loaded in indicated amounts (ng) onto non-reducing SDS-gels as standard together with 1.6 µg or 0.8 µg of processed BMP-10 CPLX followed by SDS-PAGE. The gel was stained with Coomassie to quantify the GF amounts within the BMP-10 CPLX samples.

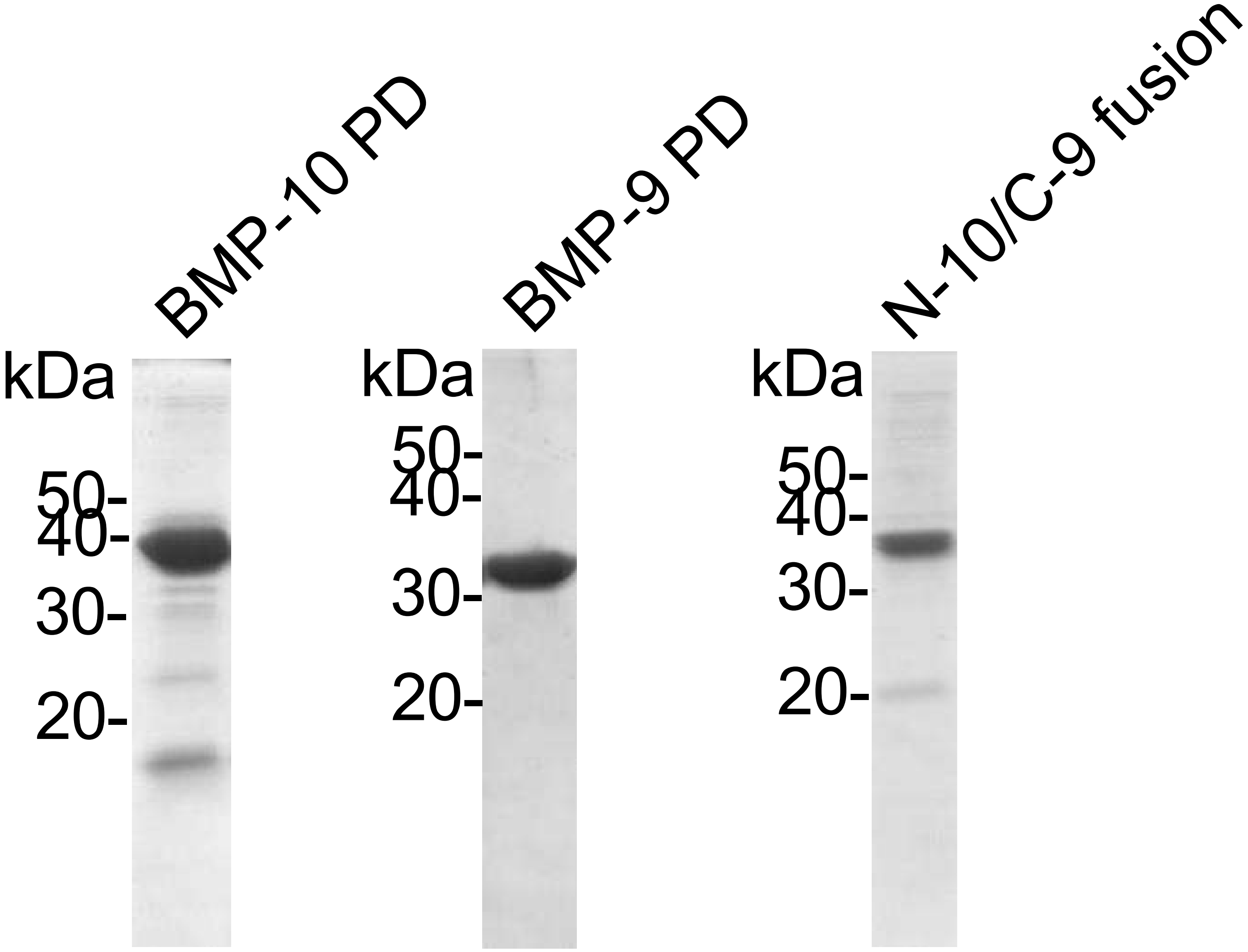

Figure S3: Coomassie-stained reducing gel of BMP-10 PD, BMP-9 PD and N-10/C-9 fusion PD expressed with a C-terminally placed His_6-tag in *E. coli.*

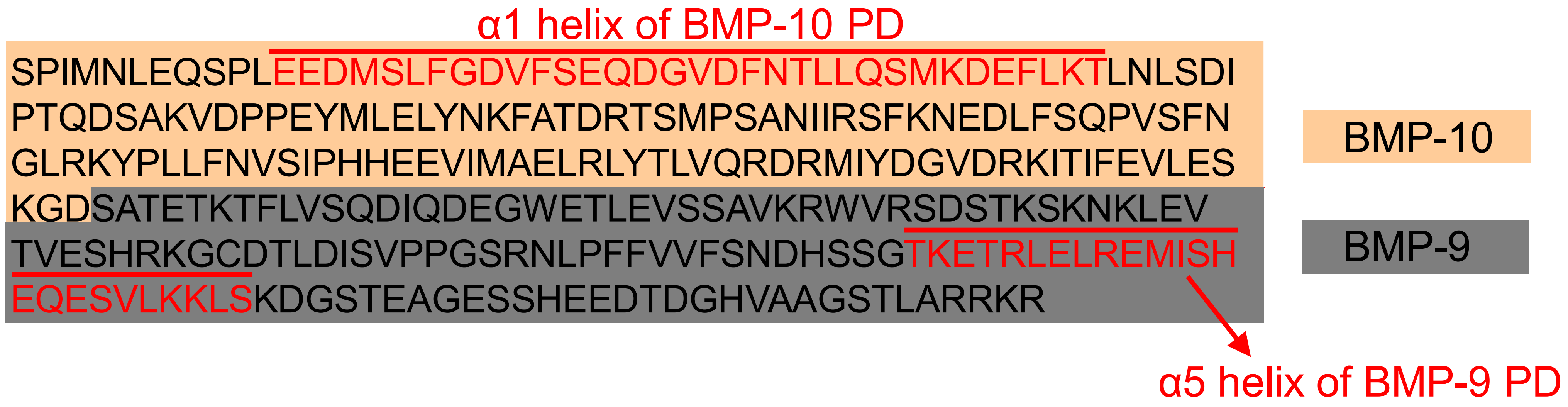

Figure S4: Amino acid sequence of the human N-10/C-9 fusion.

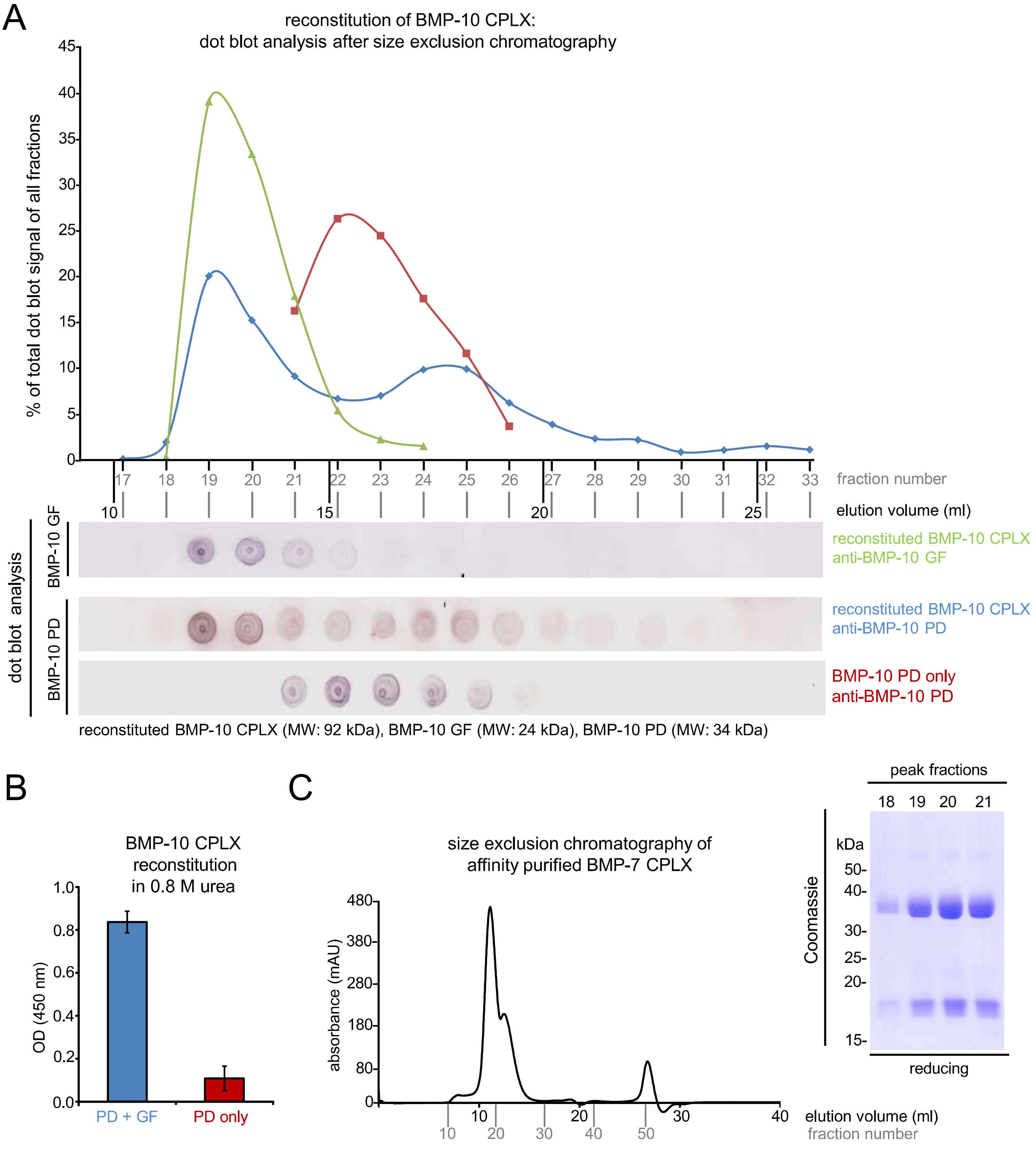

Figure S5: Reconstitution of BMP-10 CPLX by dialyzation of PD (*E coli.* expressed) together with GF dimer (commercially available, overexpressed in HEK293 cell culture). (A) Dot blot analysis of size exclusion chromatography (SEC) elution fractions after reconstitution (molar ratio PD:GF at 3:1) showed presence of reconstituted BMP-10 CPLX in fractions 19-21 as indicated by parallel detection of BMP-10 PD and GF signals. SEC of purified BMP-10 PD only served as reference. (B) Sandwich ELISA detection of reconstituted BMP-10 CPLX (in HEPES buffer/ 0.8 M urea in presence of 0.1% BSA) using anti-BMP-10 GF antibody as capture and anti-BMP-10 PD antibody as detector. (C) SEC of BMP-7 CPLX after affinity purification. (left) Elution fractions showed highest absorbance in peak fractions around 11 ml. (right) SDS-PAGE analysis (reducing conditions) of peak fractions followed by Coomassie staining.

Affinity purified full length BMP-10 after overexpression in HEK293 cells using the sleeping beauty transposon system

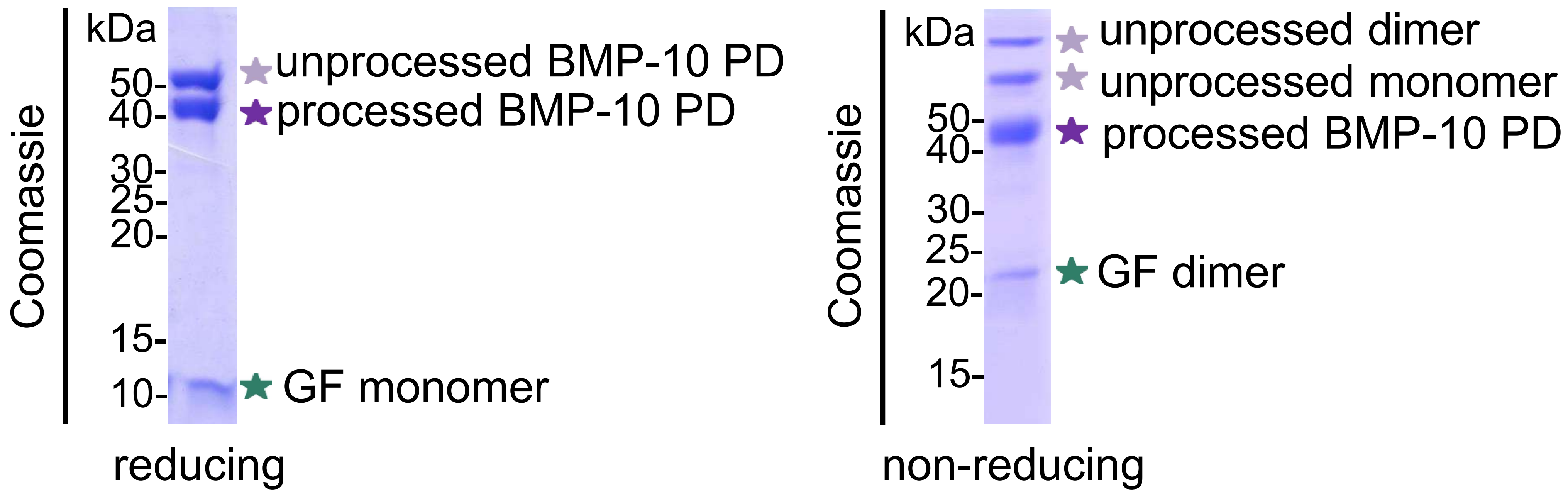

Figure S6: Overexpression of BMP-10 in HEK293 cells using the sleeping beauty transposon system yielded a 1:1 ratio of unprocessed and processed BMP-10 molecules. The sleeping beauty (SB) transposon system allows integration into the genome of HEK293 cells and induction of its production upon doxycycline treatment. Serum-free supernatant from overexpressing cells was affinity purified via a C-terminally placed His_6-tag on the PD domain right before the PPC site. The overexpression using the SB system yielded higher protein amounts but overwhelmed the PPC machinery so that BMP-10 could not be quantitatively processed anymore.

BMP-10 PD of the unprocessed BMP-10 dimer

```
Score          Expect Method                       Identities     Positives      Gaps
835 bits(2157) 0.0    Compositional matrix adjust. 401/403(99%)   401/403(99%)   0/403(0%)

uniprot 1    SPIMNLEQSPLEEDMSLFGDVFSEQDGVDFNTLLQSMKDEFLKTLNLSDIPTQDSAKVDP  60
             SPIMNLEQSPLEEDMSLFGDVFSEQDGVDFNTLLQSMKDEFLKTLNLSDIPTQDSAKVDP
model   1    SPIMNLEQSPLEEDMSLFGDVFSEQDGVDFNTLLQSMKDEFLKTLNLSDIPTQDSAKVDP  60

uniprot 61   PEYMLELYNKFATDRTSMPSANIIRSFKNEDLFSQPVSFNGLRKYPLLFNVSIPHHEEVI  120
             PEYMLELYNKFATDRTSMPSANIIRSFKNEDLFSQPVSFNGLRKYPLLFNVSIPHHEEVI
model   61   PEYMLELYNKFATDRTSMPSANIIRSFKNEDLFSQPVSFNGLRKYPLLFNVSIPHHEEVI  120

uniprot 121  MAELRLYTLVQRDRMIYDGVDRKITIFEVLESKGDNEGERNMLVLVSGEIYGTNSEWETF  180
             MAELRLYTLVQRDRMIYDGVDRKITIFEVLESKGDNEGERNMLVLVSGEIYGTNSEWETF
model   121  MAELRLYTLVQRDRMIYDGVDRKITIFEVLESKGDNEGERNMLVLVSGEIYGTNSEWETF  180

uniprot 181  DVTDAIRRWQKSGSSTHQLEVHIESKHDEAEDASSGRLEIDTSAQNKHNPLLIVFSDDQS  240
             DVTDAIRRWQKSGSSTHQLEVHIESKHDEAEDASSGRLEIDTSAQNKHNPLLIVFSDDQS
model   181  DVTDAIRRWQKSGSSTHQLEVHIESKHDEAEDASSGRLEIDTSAQNKHNPLLIVFSDDQS  240

uniprot 241  SDKERKEELNEMISHEQLPELDNLGLDSFSSGPGEEALLQMRSNIIYDSTARIRRNAKGN  300
             SDKERKEELNEMISHEQLPELDNLGLDSFSSGPGEEALLQMRSNIIYDSTAR RNAKGN
model   241  SDKERKEELNEMISHEQLPELDNLGLDSFSSGPGEEALLQMRSNIIYDSTARSIRNAKGN  300

uniprot 301  YCKRTPLYIDFKEIGWDSWIIAPPGYEAYECRGVCNYPLAEHLTPTKHAIIQALVHLKNS  360
             YCKRTPLYIDFKEIGWDSWIIAPPGYEAYECRGVCNYPLAEHLTPTKHAIIQALVHLKNS
model   301  YCKRTPLYIDFKEIGWDSWIIAPPGYEAYECRGVCNYPLAEHLTPTKHAIIQALVHLKNS  360

uniprot 361  QKASKACCVPTKLEPISILYLDKGVVTYKFKYEGMAVSECGCR  403
             QKASKACCVPTKLEPISILYLDKGVVTYKFKYEGMAVSECGCR
model   361  QKASKACCVPTKLEPISILYLDKGVVTYKFKYEGMAVSECGCR  403
```

BMP-10 PD of the processed BMP-10 CPLX

```
Score          Expect Method                       Identities     Positives      Gaps
597 bits(1538) 0.0    Compositional matrix adjust. 293/295(99%)   293/295(99%)   0/295(0%)

uniprot 1    SPIMNLEQSPLEEDMSLFGDVFSEQDGVDFNTLLQSMKDEFLKTLNLSDIPTQDSAKVDP  60
             SPIMNLEQSPLEEDMSLFGDVFSEQDGVDFNTLLQSMKDEFLKTLNLSDIPTQDSAKVDP
model   1    SPIMNLEQSPLEEDMSLFGDVFSEQDGVDFNTLLQSMKDEFLKTLNLSDIPTQDSAKVDP  60

uniprot 61   PEYMLELYNKFATDRTSMPSANIIRSFKNEDLFSQPVSFNGLRKYPLLFNVSIPHHEEVI  120
             PEYMLELYNKFATDRTSMPSANIIRSFKNEDLFSQPVSFNGLRKYPLLFNVSIPHHEEVI
model   61   PEYMLELYNKFATDRTSMPSANIIRSFKNEDLFSQPVSFNGLRKYPLLFNVSIPHHEEVI  120

uniprot 121  MAELRLYTLVQRDRMIYDGVDRKITIFEVLESKGDNEGERNMLVLVSGEIYGTNSEWETF  180
             MAELRLYTLVQRDRMIYDGVDRKITIFEVLESKGDNEGERNMLVLVSGEIYGTNSEWETF
model   121  MAELRLYTLVQRDRMIYDGVDRKITIFEVLESKGDNEGERNMLVLVSGEIYGTNSEWETF  180

uniprot 181  DVTDAIRRWQKSGSSTHQLEVHIESKHDEAEDASSGRLEIDTSAQNKHNPLLIVFSDDQS  240
             DVTDAIRRWQKSGSSTHQLEVHIESKHDEAEDASSGRLEIDTSAQNKHNPLLIVFSDDQS
model   181  DVTDAIRRWQKSGSSTHQLEVHIESKHDEAEDASSGRLEIDTSAQNKHNPLLIVFSDDQS  240

uniprot 241  SDKERKEELNEMISHEQLPELDNLGLDSFSSGPGEEALLQMRSNIIYDSTARIRR  295
             SDKERKEELNEMISHEQLPELDNLGLDSFSSGPGEEALLQMRSNIIYDSTAR  R
model   241  SDKERKEELNEMISHEQLPELDNLGLDSFSSGPGEEALLQMRSNIIYDSTARSIR  295
```

processed BMP-10 PD of the closed-ring BMP-10 CPLX

```
Score          Expect Method                       Identities     Positives      Gaps
597 bits(1538) 0.0    Compositional matrix adjust. 293/295(99%)   293/295(99%)   0/295(0%)

uniprot 1    SPIMNLEQSPLEEDMSLFGDVFSEQDGVDFNTLLQSMKDEFLKTLNLSDIPTQDSAKVDP  60
             SPIMNLEQSPLEEDMSLFGDVFSEQDGVDFNTLLQSMKDEFLKTLNLSDIPTQDSAKVDP
model   1    SPIMNLEQSPLEEDMSLFGDVFSEQDGVDFNTLLQSMKDEFLKTLNLSDIPTQDSAKVDP  60

uniprot 61   PEYMLELYNKFATDRTSMPSANIIRSFKNEDLFSQPVSFNGLRKYPLLFNVSIPHHEEVI  120
             PEYMLELYNKFATDRTSMPSANIIRSFKNEDLFSQPVSFNGLRKYPLLFNVSIPHHEEVI
model   61   PEYMLELYNKFATDRTSMPSANIIRSFKNEDLFSQPVSFNGLRKYPLLFNVSIPHHEEVI  120

uniprot 121  MAELRLYTLVQRDRMIYDGVDRKITIFEVLESKGDNEGERNMLVLVSGEIYGTNSEWETF  180
             MAELRLYTLVQRDRMIYDGVDRKITIFEVLESKGDNEGERNMLVLVSGEIYGTNSEWETF
model   121  MAELRLYTLVQRDRMIYDGVDRKITIFEVLESKGDNEGERNMLVLVSGEIYGTNSEWETF  180

uniprot 181  DVTDAIRRWQKSGSSTHQLEVHIESKHDEAEDASSGRLEIDTSAQNKHNPLLIVFSDDQS  240
             DVTDAIRRWQKSGSSTHQLEVHIESKHDEAEDASSGRLEIDTSAQNKHNPLLIVFSDDQS
model   181  DVTDAIRRWQKSGSSTHQLEVHIESKHDEAEDASSGRLEIDTSAQNKHNPLLIVFSDDQS  240

uniprot 241  SDKERKEELNEMISHEQLPELDNLGLDSFSSGPGEEALLQMRSNIIYDSTARIRR  295
             SDKERKEELNEMISHEQLPELDNLGLDSFSSGPGEEALLQMRSNIIYDSTAR  R
model   241  SDKERKEELNEMISHEQLPELDNLGLDSFSSGPGEEALLQMRSNIIYDSTARSIR  295
```

BMP-10 PD of the BMP-10 CPLX atomic model (PDB: 7POI)

```
Score         Expect Method                       Identities     Positives      Gaps
272 bits(696) 2e-98  Compositional matrix adjust. 143/182(79%)   143/182(78%)   39/182(21%)

model   1    VDPPEYMLELYNKFATDRTSMPSANIIRSFKNEDLFSQPVSFNGLRKYPLLFNVSIPHHE  60
             VDPPEYMLELYNKFATDRTSMPSANIIRSFKNEDLFSQPVSFNGLRKYPLLFNVSIPHHE
uniprot 58   VDPPEYMLELYNKFATDRTSMPSANIIRSFKNEDLFSQPVSFNGLRKYPLLFNVSIPHHE  117

model   61   EVIMAELRLY-------------DRKITIFEVL------EGERNMLVLVSGEIYGTNSEW  101
             EVIMAELRLY             DRKITIFEVL      EGERNMLVLVSGEIYGTNSEW
uniprot 118  EVIMAELRLYTLVQRDRMIYDGVDRKITIFEVLESKGDNEGERNMLVLVSGEIYGTNSEW  177

model   102  ETFDVTDAIRRWQKSGSSTHQLEVHIES--------------------NKHNPLLIVFSD  141
             ETFDVTDAIRRWQKSGSSTHQLEVHIES                    NKHNPLLIVFSD
uniprot 178  ETFDVTDAIRRWQKSGSSTHQLEVHIESKHDEAEDASSGRLEIDTSAQNKHNPLLIVFSD  237

model   142  DQ  143
             DQ
uniprot 238  DQ  239
```

BMP-10 GF of the closed-ring BMP-10 CPLX

```
Score         Expect Method                       Identities      Positives       Gaps
225 bits(574) 1e-83  Compositional matrix adjust. 108/108(100%)   108/108(100%)   0/108(0%)

model   1    NAKGNYCKRTPLYIDFKEIGWDSWIIAPPGYEAYECRGVCNYPLAEHLTPTKHAIIQALV  60
             NAKGNYCKRTPLYIDFKEIGWDSWIIAPPGYEAYECRGVCNYPLAEHLTPTKHAIIQALV
uniprot 1    NAKGNYCKRTPLYIDFKEIGWDSWIIAPPGYEAYECRGVCNYPLAEHLTPTKHAIIQALV  60

model   61   HLKNSQKASKACCVPTKLEPISILYLDKGVVTYKFKYEGMAVSECGCR  108
             HLKNSQKASKACCVPTKLEPISILYLDKGVVTYKFKYEGMAVSECGCR
uniprot 61   HLKNSQKASKACCVPTKLEPISILYLDKGVVTYKFKYEGMAVSECGCR  108
```

BMP-10 GF of the BMP-10 CPLX atomic model (PDB: 7POI)

```
Score         Expect Method                       Identities      Positives       Gaps
218 bits(554) 1e-80  Compositional matrix adjust. 104/104(100%)   104/104(100%)   0/104(0%)

model   1    NYCKRTPLYIDFKEIGWDSWIIAPPGYEAYECRGVCNYPLAEHLTPTKHAIIQALVHLKN  60
             NYCKRTPLYIDFKEIGWDSWIIAPPGYEAYECRGVCNYPLAEHLTPTKHAIIQALVHLKN
uniprot 5    NYCKRTPLYIDFKEIGWDSWIIAPPGYEAYECRGVCNYPLAEHLTPTKHAIIQALVHLKN  64

model   61   SQKASKACCVPTKLEPISILYLDKGVVTYKFKYEGMAVSECGCR  104
             SQKASKACCVPTKLEPISILYLDKGVVTYKFKYEGMAVSECGCR
uniprot 65   SQKASKACCVPTKLEPISILYLDKGVVTYKFKYEGMAVSECGCR  108
```

BMP-10 GF of the processed BMP-10 CPLX

```
Score         Expect Method                       Identities      Positives       Gaps
225 bits(574) 1e-83  Compositional matrix adjust. 108/108(100%)   108/108(100%)   0/108(0%)

model   1    NAKGNYCKRTPLYIDFKEIGWDSWIIAPPGYEAYECRGVCNYPLAEHLTPTKHAIIQALV  60
             NAKGNYCKRTPLYIDFKEIGWDSWIIAPPGYEAYECRGVCNYPLAEHLTPTKHAIIQALV
uniprot 1    NAKGNYCKRTPLYIDFKEIGWDSWIIAPPGYEAYECRGVCNYPLAEHLTPTKHAIIQALV  60

model   61   HLKNSQKASKACCVPTKLEPISILYLDKGVVTYKFKYEGMAVSECGCR  108
             HLKNSQKASKACCVPTKLEPISILYLDKGVVTYKFKYEGMAVSECGCR
uniprot 61   HLKNSQKASKACCVPTKLEPISILYLDKGVVTYKFKYEGMAVSECGCR  108
```

Figure S7: Amino acid sequence coverage of BMP-10 models used in this study.

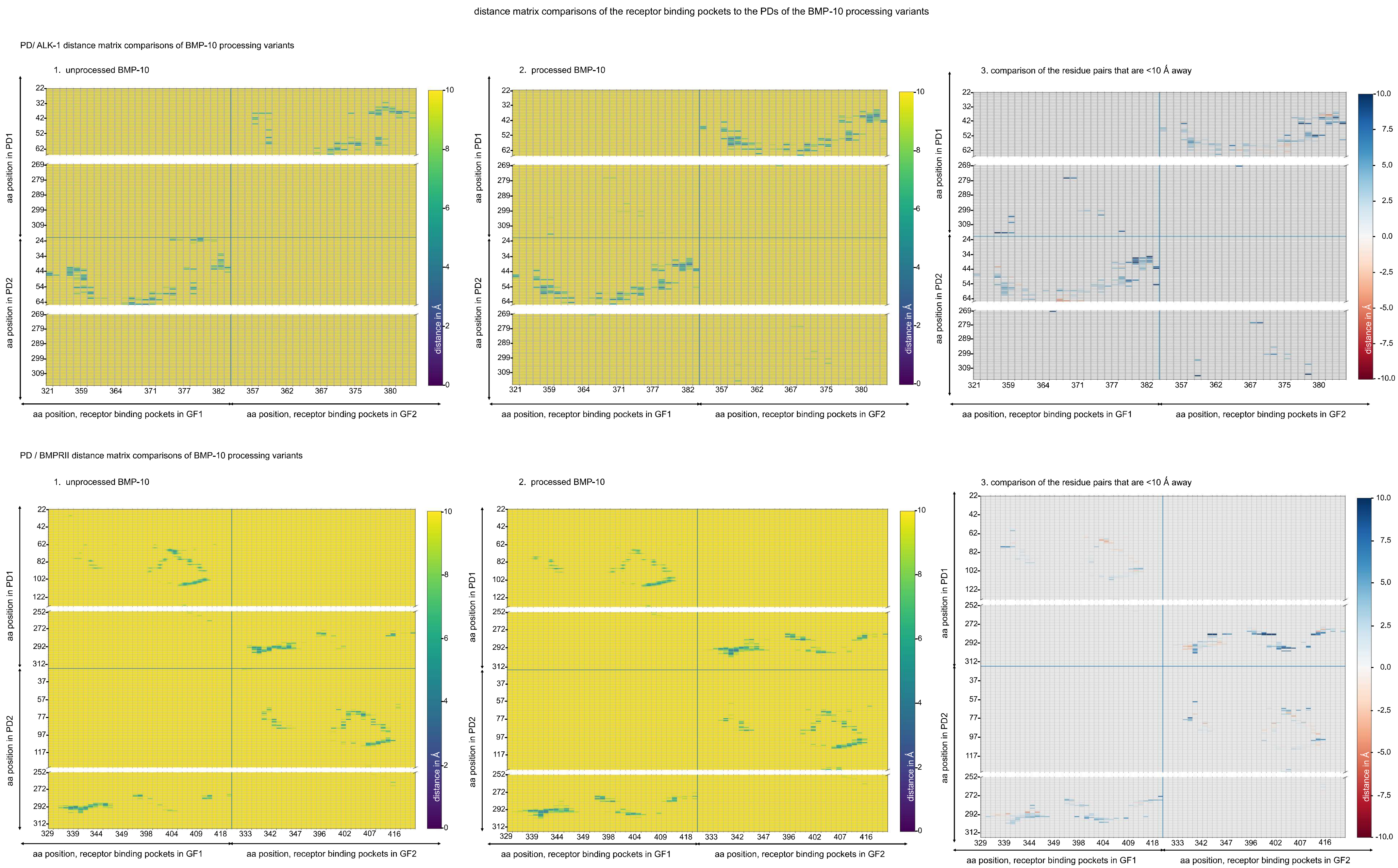

Figure S8: Distance matrix comparisons of the receptor binding pockets in the growth factor to the prodomains of the unprocessed and processed BMP-10. The distance in Å was measured between the already characterized ALK-1 and BMPRII receptor binding pockets in the BMP-10 GF (63) and the PDs of the BMP-10 processing variants.

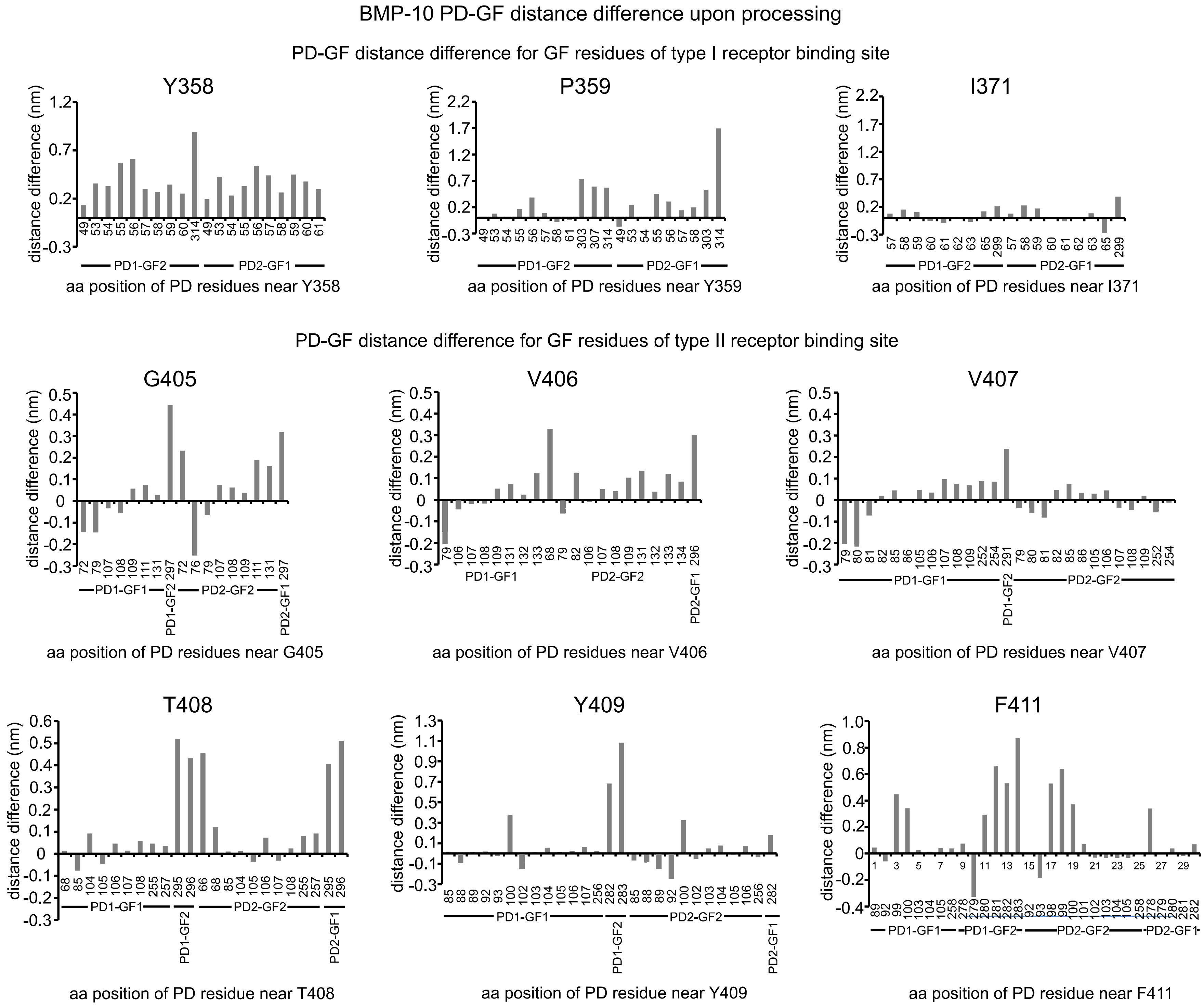

Figure S9: Distance measurements of PD residues near GF residues constituting BMP type I and II receptor binding sites show overall increase upon processing. Positive values indicate an increased exposure of GF residues in the processed BMP-10 model in comparison to the unprocessed BMP-10 model. Negative values indicate less exposure of GF residues in the processed BMP-10 model in comparison to the unprocessed BMP-10 model.

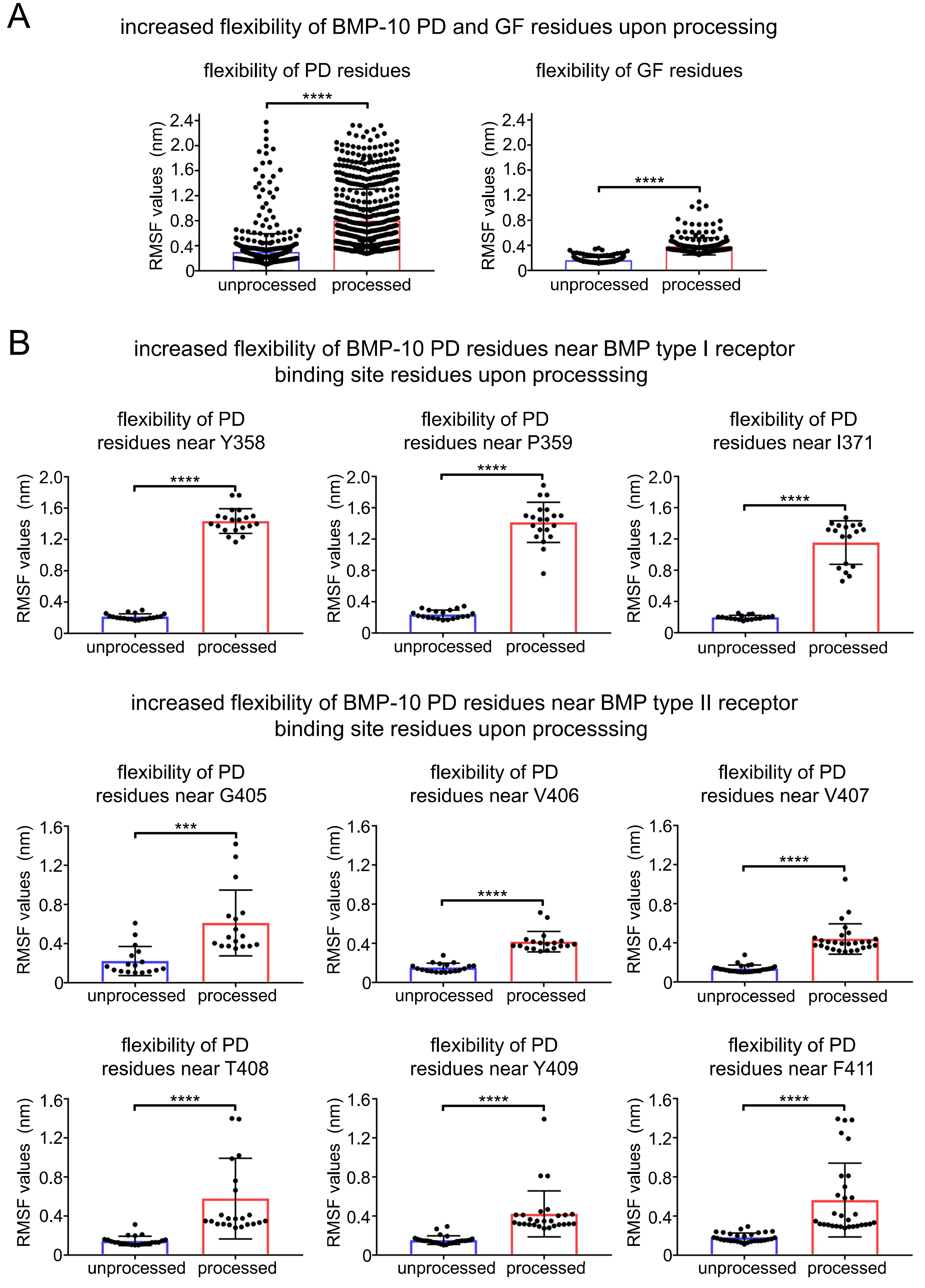

Figure S10: Root mean square fuctuation (RMSF) values derived from molecular dynamics simulations indicate a significant increase in flexibility of BMP-10 PD and GF residues upon processing. (A) All BMP-10 PD and GF residues show a significant increase in RMSF values upon processing, indicating an increase of flexibility. (B) PDs residues near BMP type I and II receptor binding site residues within the GF show a significant increase in flexibility upon BMP-10 processing.

BMP-10 PDs self-interact to build a stable PD dimer clamp under physiological conditions

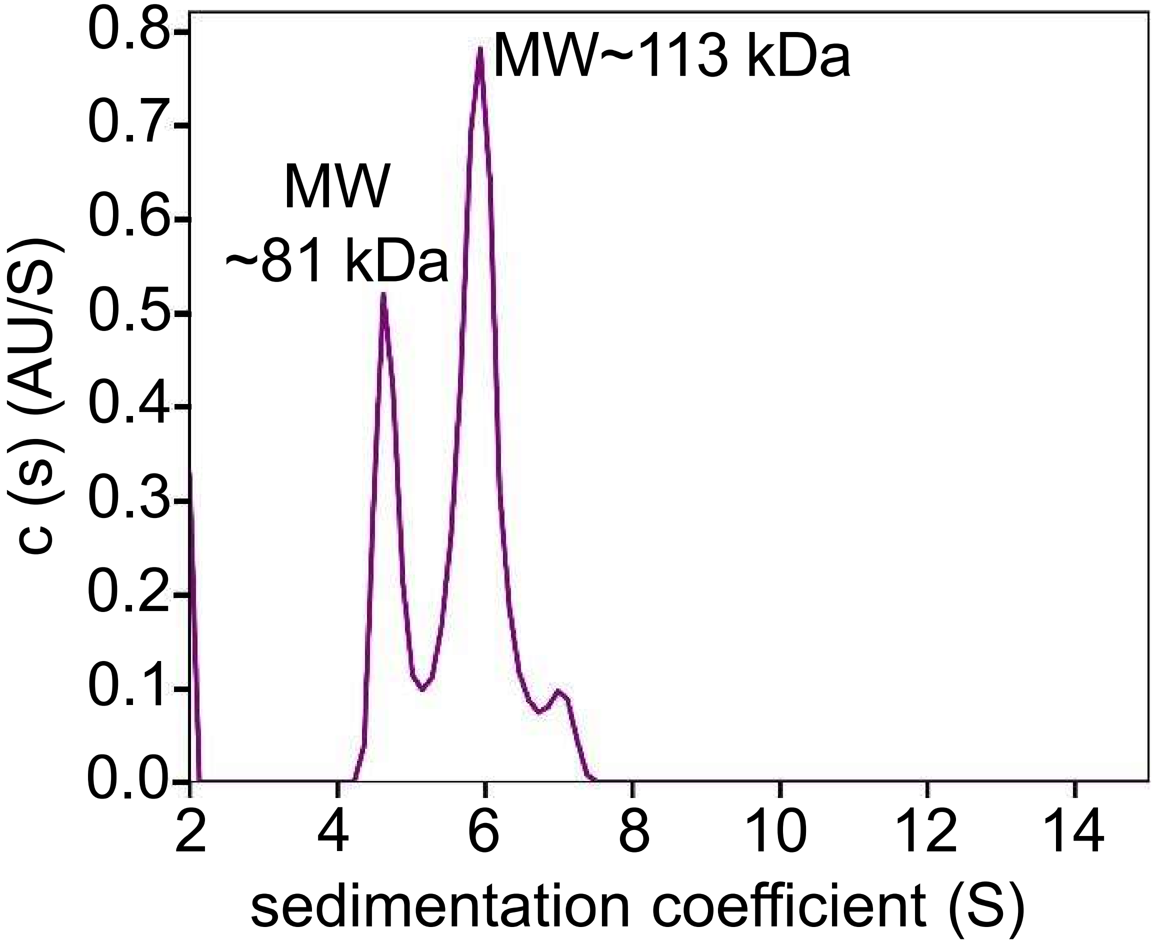

Figure S11: Presence of BMP-10 PD dimer in processed BMP-10 CPLX sample. Analytical ultracentrifugation (AUC) after SEC/ MALS of the processed BMP-10 CPLX shows clear peaks at the sizes of a PD dimer (81 kDa) and BMP-10 CPLX (113 kDa) respectively.

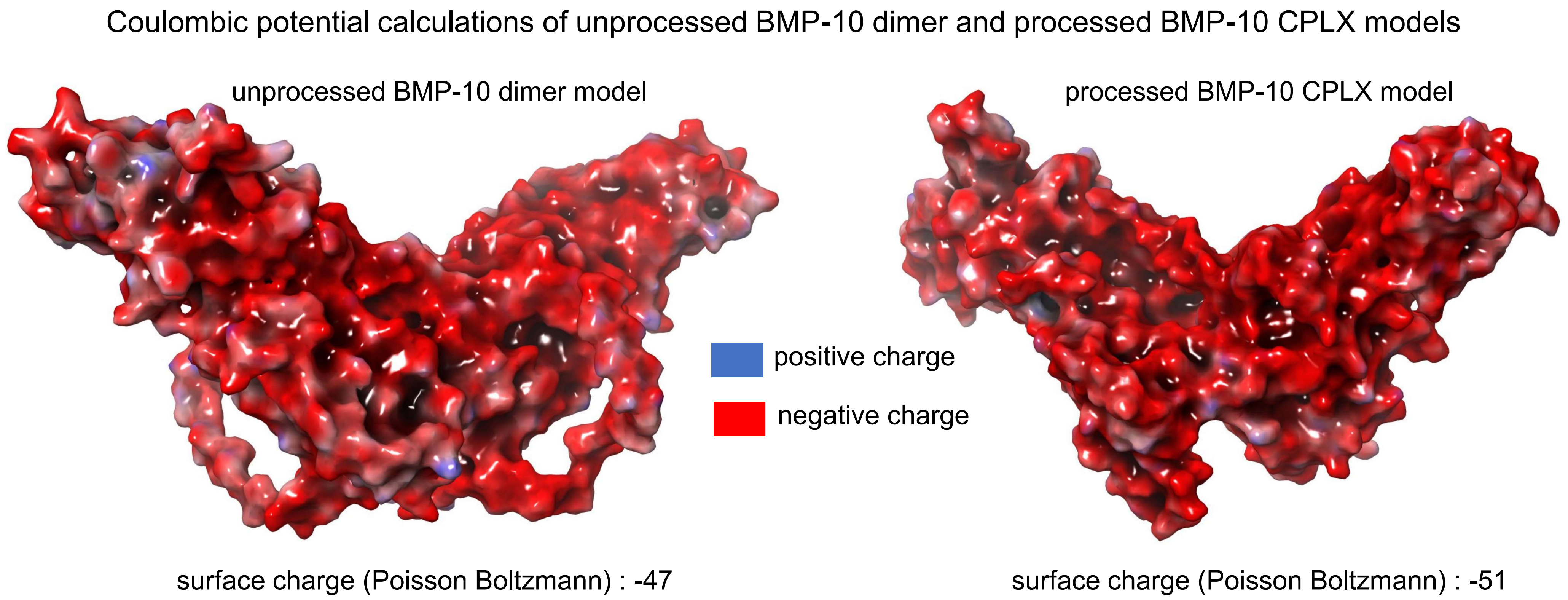

Fig. S12: Surface charge calculations (Poisson Boltzmann) of unprocessed BMP-10 or processed BMP-10 CPLX models at pH 8.4.

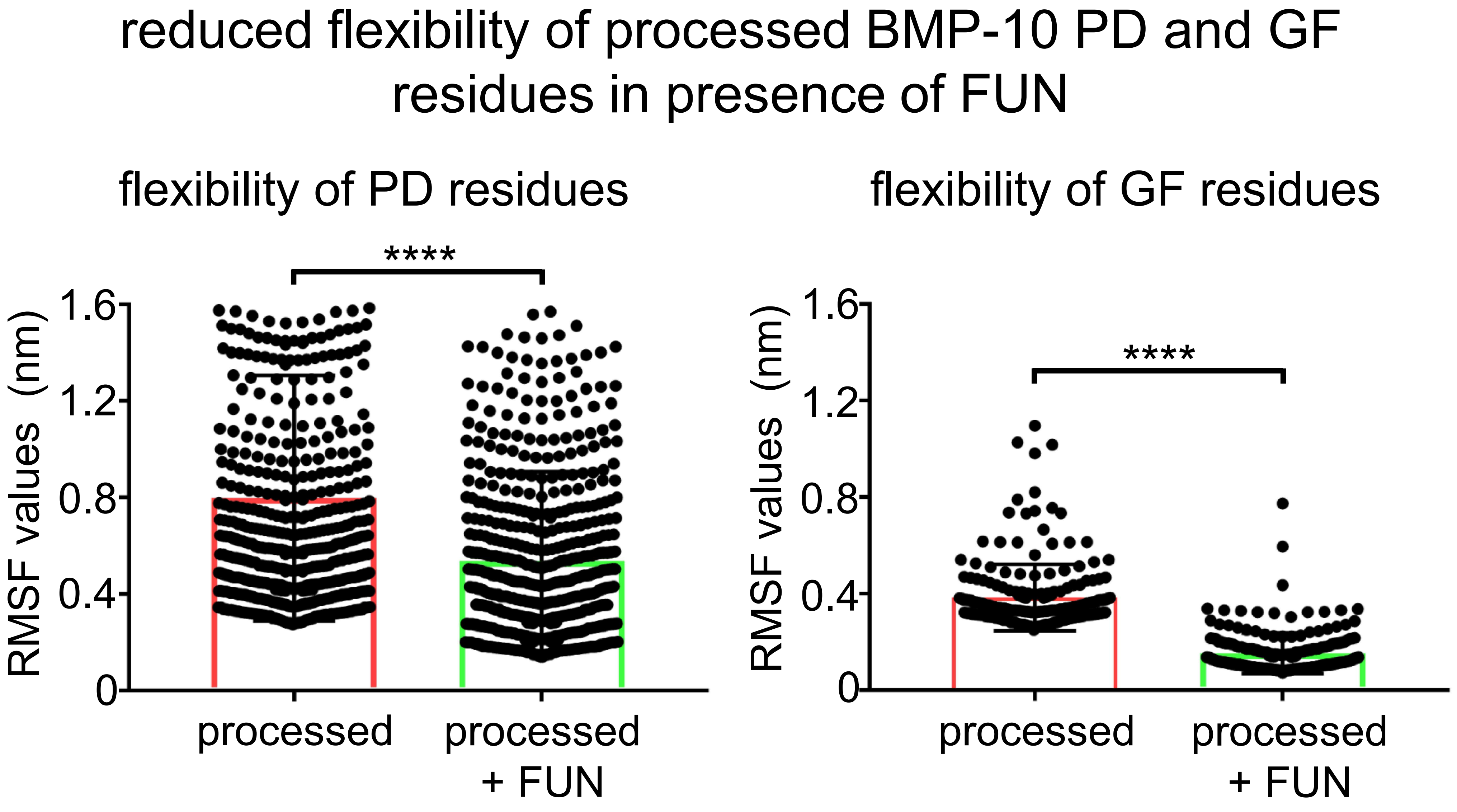

Figure S13: Root mean square fluctuation (RMSF) values derived from molecular dynamics simulations (shown in supplemental video 1 and 2) indicate significantly reduced flexibility of processed BMP-10 PD and GF residues in the presence of the fibrillin-1 FUN domain.
